# Integrated Multi‐Omics Reveals Cellular States and Microenvironmental Remodeling in Coexisting DCIS and IDC

**DOI:** 10.1002/advs.77897

**Published:** 2026-09-27

**Authors:** Ning Zhang, Tong Wan, Siyue Zhang, Yunzhen Jiang, Peng Su, Jixin Liu, Zhitong Chen, Jie Hao, Haoyu Wang, Bing Chen, Wenjing Zhao, Lijuan Wang, Tao Xing, Qihuang Zhang, Ulf Schmitz, Zhiyong Ding, Nicola Crosetto, Bingqiang Liu, Qifeng Yang

**Affiliations:** ^1^ Department of Breast Surgery General Surgery Qilu Hospital of Shandong University Ji'nan China; ^2^ Department of General Surgery Beijing Tiantan Hospital Capital Medical University Beijing China; ^3^ Cheong Kun Lun College University of Macau Macau China; ^4^ Department of Pathology Qilu Hospital of Shandong University Ji'nan China; ^5^ School of Mathematics Shandong University Ji'nan China; ^6^ Biological Resource Center Qilu Hospital of Shandong University Ji'nan China; ^7^ Department of Radiation Oncology Townsville University Hospital Townsville Australia; ^8^ College of Medicine and Dentistry James Cook University Townsville Australia; ^9^ Department of Epidemiology Biostatistics and Occupational Health School of Population and Global Health McGill University Montreal Canada; ^10^ Computational Biomedicine Lab College of Science and Engineering James Cook University Townsville Australia; ^11^ Centre for Tropical Bioinformatics and Molecular Biology James Cook University Cairns Australia; ^12^ Centenary Institute The University of Sydney Camperdown Australia; ^13^ Mills Institute for Personalized Cancer Care Fynn Biotechnologies Ltd. Ji'nan China; ^14^ Department of Microbiology Tumor and Cell Biology Karolinska Institutet Stockholm Sweden; ^15^ Science for Life Laboratory Stockholm Sweden; ^16^ Human Technopole Milan Italy; ^17^ Research Institute of Breast Cancer Shandong University Ji'nan China

**Keywords:** ductal carcinoma in situ, invasive ductal carcinoma, machine learning, multi‐omics integration, single‐cell sequencing, spatial transcriptomics

## Abstract

Ductal carcinoma in situ (DCIS) is a non‐invasive precursor of invasive ductal carcinoma (IDC), yet the biological mechanisms underlying the transition from DCIS to IDC remain incompletely understood. Here, we integrate spatial transcriptomics, single‐cell RNA sequencing, and single‐cell DNA sequencing on coexisting DCIS and IDC samples to characterize cellular and microenvironmental alterations. Integrated analyses reveal differential molecular characterizations between coexisting DCIS and IDC and identify candidate genes (*MGP*, *PLAT*, and *SERPINA3*) potentially limiting the progression from DCIS to IDC. Malignant epithelial meta‐programs (MPs) delineate distinct transcriptional states, with development‐associated MP1 enriched in DCIS and cell cycle‐related MP5 enriched in IDC. Further analysis reveals varied microenvironmental features in DCIS and IDC, with invasion‐associated Mph_SPP1 and development‐associated iCAFs_HOPX enriched in DCIS, while immunosuppressive Mph_PRDM1 and metabolism‐related tCAFs_BNIP3 predominate in IDC. We then construct a machine learning model to identify DCIS at high risk of progression, which is externally validated across independent bulk RNA‐sequencing cohorts (mean AUC = 0.903). Collectively, this study provides an integrative framework for understanding the molecular and spatial features that distinguish DCIS from IDC, with the potential to inform more precise risk stratification and clinical management for DCIS progression.

## Graphical abstract

Coexisting DCIS and IDC samples are profiled using spatial transcriptomics, single‐cell RNA sequencing, and single‐cell DNA sequencing. Integrative multi‐omics analysis reveals distinct malignant epithelial and microenvironmental features between DCIS and IDC, which are further validated using Xenium and multiplex immunohistochemistry. These features are subsequently incorporated into a machine‐learning model to identify high‐risk DCIS subgroups.

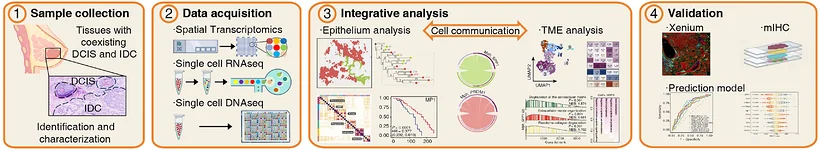

## 1 Introduction

The widespread implementation of preventive breast cancer screening has significantly improved the early detection of mammary gland neoplasms [1]. Ductal carcinoma in situ (DCIS), a non‐invasive precursor to invasive ductal carcinoma (IDC), now accounts for approximately 20% of newly diagnosed breast cancer cases [2]. Standard treatment of DCIS typically involves mastectomy or breast‐conserving surgery followed by radiotherapy, despite increasing evidence that only a subset of lesions progress to invasive disease over a patient's lifetime. This potential overtreatment is largely attributable to our incomplete understanding of the biological mechanisms driving the progression from DCIS to IDC [3, 4, 5], which limits the ability to reliably distinguish indolent from high‐risk lesions using predictive molecular indicators.

To address this, several studies have employed omics‐based approaches to investigate the molecular differences between DCIS and IDC, primarily using metachronous samples collected at different time points [2, 6, 7, 8, 9]. Nevertheless, the progression from DCIS to IDC demonstrates considerable variability, influenced by tumor heterogeneity and complex interactions within the tumor microenvironment (TME) at both genomic and transcriptomic levels [10, 11]. Accordingly, comparative analyses of DCIS and IDC using synchronous samples that share a common genetic background and thereby minimize confounding factors, combined with high‐resolution, multi‐omics profiling, are essential for identifying molecular signatures predictive of progression from in situ to invasive disease.

Recent advances in spatial omics approaches, such as spatial transcriptomics (ST), have enabled region‐resolved profiling of coexisting pathological types within intact tissue sections, thereby allowing synchronous lesions to be delineated without loss of spatial context [12]. However, ST is generally constrained by limited spatial resolution and often captures mixed signals from multiple cells, making it difficult to resolve cellular states at single‐cell resolution [13]. Single‐cell sequencing can complement this limitation by providing high‐resolution cellular and genomic information. Given that the progression from DCIS to IDC involves both transcriptional reprogramming and genomic alterations, integrating single‐cell RNA sequencing (scRNAseq) and single‐cell DNA sequencing (scDNAseq) with ST is essential for delineating the cellular, spatial, and genetic heterogeneity underlying this transition [14, 15, 16]. Such cellularly and spatially informed features may also provide a biologically grounded basis for downstream risk modeling. Indeed, the integration of multi‐omics analysis with machine learning algorithms has been successfully applied to predictive model construction in various diseases [17]. Although several studies have attempted to identify biomarkers for predicting DCIS progression risk [18, 19, 20], approaches that derive progression‐related features and systematically evaluate them across multiple machine learning algorithms for DCIS progression risk prediction remain limited.

Here, we conducted a comprehensive multi‐omics study, incorporating ST, scRNAseq, and scDNAseq on coexisting DCIS and IDC samples to investigate the biological distinctions and mechanisms underlying DCIS‐to‐IDC progression. This integrative approach enabled simultaneous profiling of transcriptional and genomic features across malignant epithelial cells and the TME. Using ST, we first characterized spatial gene expression patterns and functional pathway differences between DCIS and IDC. By integrating our previously developed scDNAseq method, scCUTseq [21], we reconstructed copy number variation (CNV)‐based phylogenetic trees and compared matched DCIS and IDC subclones to identify invasion associated transcriptional changes and genetic alterations. Subsequent integration with scRNAseq revealed distinct malignant epithelial programs, with meta program 1 (MP1, Development related) preferentially associated with DCIS and meta program 5 (MP5, Cell Cycle related) in IDC. This analysis also identified distinct spatial distribution patterns of several TME cell populations, particularly macrophages and cancer‐associated fibroblasts (CAFs), which may contribute to the differing biological characteristics of DCIS and IDC. Finally, leveraging features derived from our multi‐omics findings, we employed machine learning models using bulk RNAseq datasets to evaluate DCIS progression risk. Collectively, our study provides an integrative and multidimensional framework for understanding the molecular, genomic, and spatial features that distinguish DCIS from IDC, offering potential insights for the development of diagnostic and therapeutic strategies for DCIS progression.

## 2 Results

### 2.1 Spatial Transcriptomics Reveals Distinct Molecular Features of Coexisting DCIS and IDC

**FIGURE 1:**
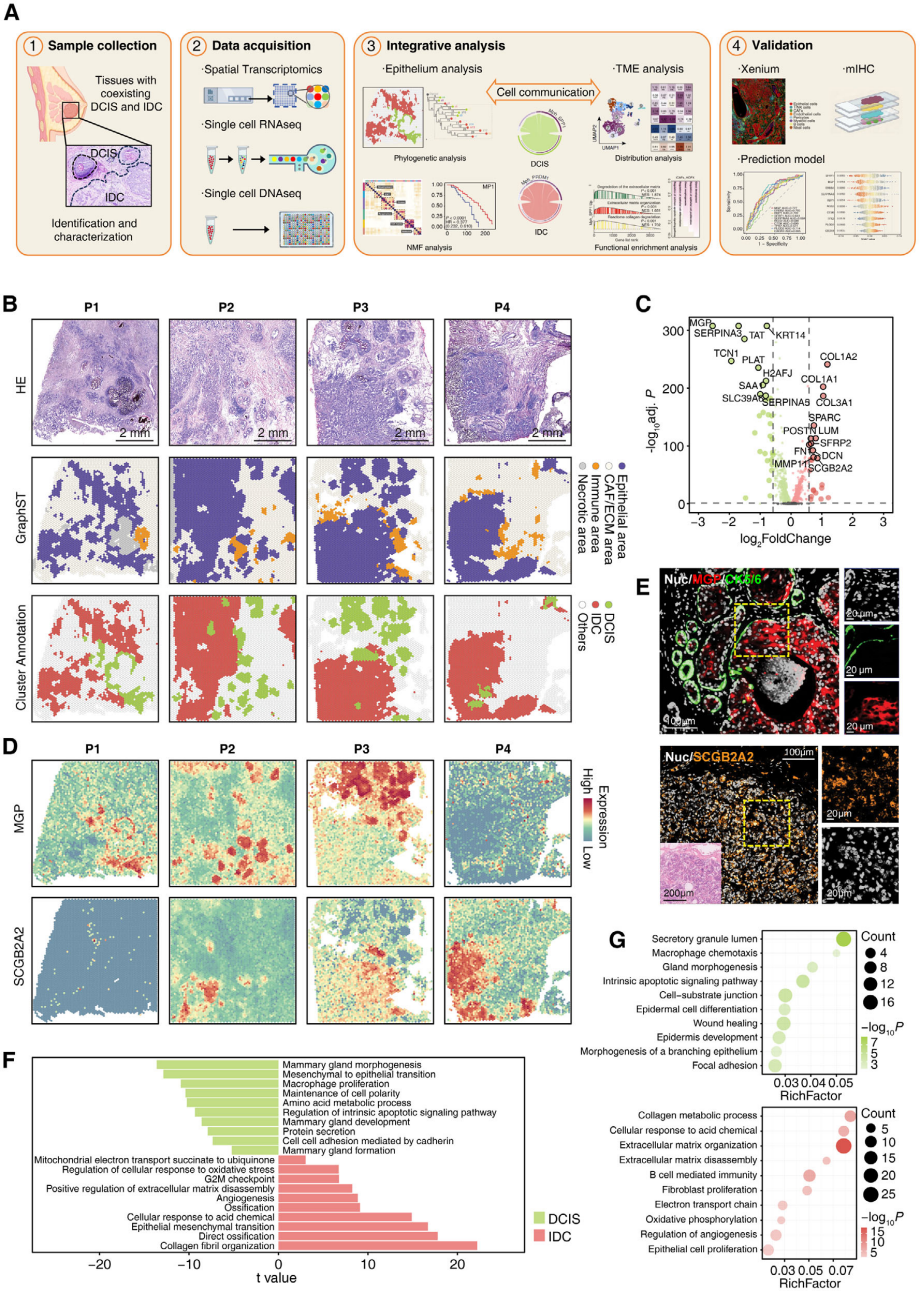
Spatial transcriptomic profiling of samples with coexisting DCIS and IDC. (A) Schematic overview of the study. Samples with coexisting DCIS and IDC were collected and processed using ST, scRNAseq and scCUTseq, followed by integrative analysis. The findings were validated by Xenium and mIHC, and a prediction model was established to evaluate the invasive‐like score of DCIS. (B) H&E‐stained tissue sections for four samples with coexisting DCIS and IDC (P1–P4) (the first row). Spot annotations based on GraphST clustering (the second row). DCIS and IDC regions defined by integrating GraphST‐derived clusters with manual pathological annotation (the third row). (C) DEGs between annotated DCIS and IDC regions in ST data, with the top 10 genes highlighted by black circles. (D) Spatial distribution of *MGP* and *SCGB2A2* in tissue sections. (E) Representative mIHC staining images of MGP and CK5/6 in DCIS and SCGB2A2 in IDC. (F) GSVA of DCIS and IDC in ST. (G) GO pathway enrichment analysis for DCIS (top panel) and IDC (bottom panel) in ST.

To characterize the heterogeneity of malignant cells and the tumor microenvironment across DCIS and IDC, breast tumor specimens were collected from patients with pathologically confirmed coexisting DCIS and IDC who underwent surgical resection at Qilu Hospital of Shandong University, China (Table S1). The specimens were first profiled using spatial transcriptomics on the 10x Visium platform, followed by scDNAseq (scCUTseq) and scRNAseq (10x Chromium) to further resolve tumor heterogeneity at single‐cell level (Figure 1A). In the initial spatial analysis, 17 079 spots from four samples were partitioned into four major spatial regions using GraphST: epithelial area, CAF/ECM‐enriched area, immune‐enriched area, and necrotic area. The GraphST‐defined epithelial areas were subsequently subdivided into DCIS and IDC regions according to pathological annotations (Figure 1B and Figure S1A).

To compare gene expression profiles between coexisting DCIS and IDC lesions, we performed differential gene expression (DEG) analysis on ST datasets. A total of 32 genes were significantly upregulated in DCIS, whereas 23 genes were significantly upregulated in IDC (Figure 1C). By integrating DEG analysis based on pathological annotations (Figure S1B), matrix Gla protein (*MGP*) and secretoglobin family 2A member 2 (*SCGB2A2*) emerged as representative epithelial marker genes for DCIS and IDC, respectively (Figure 1D). Multiplex immunohistochemistry (mIHC) further validated the preferential expression of MGP in DCIS and SCGB2A2 in IDC (Figure 1E). Gene set variation analysis (GSVA) revealed distinct biological programs between DCIS and IDC. DCIS was characterized by the enrichment of pathways associated with gland development, cell adhesion, apoptosis, and immune‐related responses, whereas IDC showed preferential enrichment of cellular respiration, epithelial–mesenchymal transition (EMT), cell cycle, and extracellular matrix disassembly pathways (Figure 1F). These findings were further supported by Gene Ontology (GO) analysis based on both GraphST‐derived and manual pathological annotations of the ST data (Figure 1G and Figure S1C), highlighting the distinct biological signatures of DCIS and IDC.

### 2.2 CNV‐Associated Clonal Evolution and Transcriptional Divergence During DCIS‐to‐IDC Progression

**FIGURE 2:**
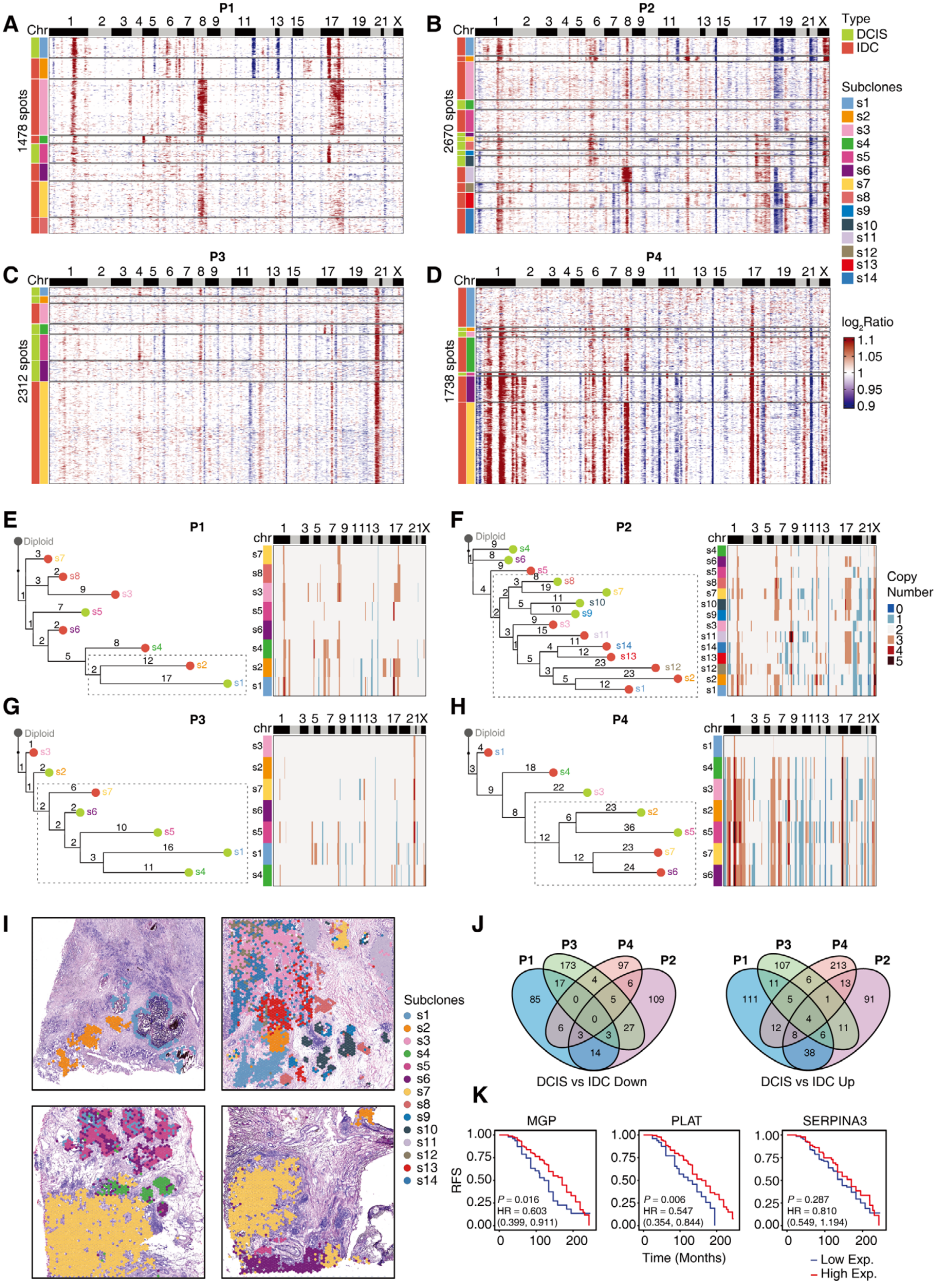
Clonal substructure and DEGs of coexisting DCIS and IDC from spatial transcriptomics. (A–D) Heatmaps of inferred CNV profiles for individual spots (rows) from ST across four samples. The left annotation columns indicate pathological classification and subclonal groups. (E–H) Left panels: Event‐based evolutionary trees of subclonal consensus integer CNV profiles, rooted in a diploid profile. IDC‐associated subclones are highlighted in red, whereas DCIS‐associated subclones are shown in green. Right panels: Heatmaps showing subclonal consensus integer CNV profiles, with the left annotation bar representing subclone identities. Dashed boxes indicate representative selected clones. Chromosome Y was excluded. (I) Spatial distribution of representative selected clones in ST. (J) Venn diagram indicates the overlap of DEGs between DCIS and IDC clones with common ancestors across four samples. (K) Kaplan–Meier survival curves showing RFS outcomes stratified by *MGP*, *PLAT*, and *SERPINA3* expression in the HTAN database. Significance was assessed using the log‐rank test, HR and 95% CI were estimated using univariable Cox proportional hazards regression.

CNVs are key genomic alterations that drive clonal evolution during the transition from DCIS to IDC, leading to the emergence of distinct CNV lineages [22]. To investigate the genomic landscape underlying this transition, we inferred genome‐wide CNV profiles from ST data using inferCNV. After filtering out low‐quality spots, 1478, 2670, 2312, and 1738 spots from the four samples were retained for downstream analysis. Unsupervised hierarchical clustering identified 7–14 CNV‐defined subclones per sample (Figure 2A–D). We next reconstructed clonal phylogenetic trees based on ST‐inferred CNV profiles to further resolve the evolutionary relationships between DCIS and IDC (Figure 2E–H), following a previously described approach [6]. Notably, highly similar subclones were identified between paired DCIS and IDC regions within each of the four samples, suggesting a shared evolutionary origin between the in situ and invasive components. For each sample, the phylogenetic branch containing the closest admixture of DCIS and IDC spots was selected and defined as the matched subclones for subsequent analyses. Spatial mapping revealed that several matched subclones were confined to discrete tumor regions, suggesting localized clonal expansion (Figure 2I).

In parallel, we applied our previously developed single‐cell DNA sequencing method, scCUTseq, to directly validate CNVs at the single‐cell whole‐genome level. After filtering out low‐quality and diploid normal cells using established pipelines, 466, 554, 640, and 458 high‐quality tumor cells were retained from the four samples, respectively (Figure S2A). Quality control metrics confirmed consistent sequencing depth and concordance with previous benchmarks, with no significant differences observed among samples (Figure S2B,C). Tumor cells were then grouped into 2–10 subclones per sample (Figure S3A–D). By calculating similarity scores between ST‐inferred CNV profiles and scCUTseq‐derived subclones, we identified the scCUTseq subclone most closely related to each ST‐defined subclone (Figure S3E–H). The concordance between the two modalities indicates that their integration provides a more robust and comprehensive depiction of CNV patterns in both DCIS and IDC. Based on this cross‐modality mapping, we further identified IDC‐specific CNV events. Among these events, amplifications of chr4q2, chr6q2 and chr12q2, together with deletion of chr13q3, were significantly associated with worse recurrence‐free survival (RFS) in the Human Tumor Atlas Network (HTAN) DCIS cohort (Figure S4A–D), suggesting that these CNV events may be involved in DCIS‐to‐IDC progression.

We further compared gene expression profiles between matched DCIS and IDC subclones to identify transcriptional changes accompanying clonal evolution during the DCIS‐to‐IDC transition. No genes were consistently upregulated in IDC across all four samples, suggesting marked inter‐patient heterogeneity in IDC‐associated transcriptional programs. In contrast, four genes, *MGP*, *PLAT*, *SERPINA3*, and *KRT14*, were consistently upregulated in DCIS relative to matched IDC subclones (Figure 2J). Because *KRT14* is a canonical basal/myoepithelial marker and may partly reflect myoepithelial components that distinguish DCIS from IDC, we focused on the remaining three genes. Prognostic analysis showed that high expression of *MGP* (HR = 0.603, 95% CI: 0.399–0.911) and *PLAT* (HR = 0.547, 95% CI: 0.354–0.884) was associated with better RFS in the HTAN dataset. *SERPINA3* showed a similar protective trend, although the association did not reach statistical significance (HR = 0.810, 95% CI: 0.549–1.194) (Figure 2K). Together, the comparison of matched DCIS and IDC subclones identified *MGP*, *PLAT*, and *SERPINA3* as DCIS‐retained genes that may contribute to limiting progression toward invasive disease.

### 2.3 Malignant Epithelial Meta‐Programs Delineate Transcriptional States Underlying DCIS‐to‐IDC Progression

**FIGURE 3:**
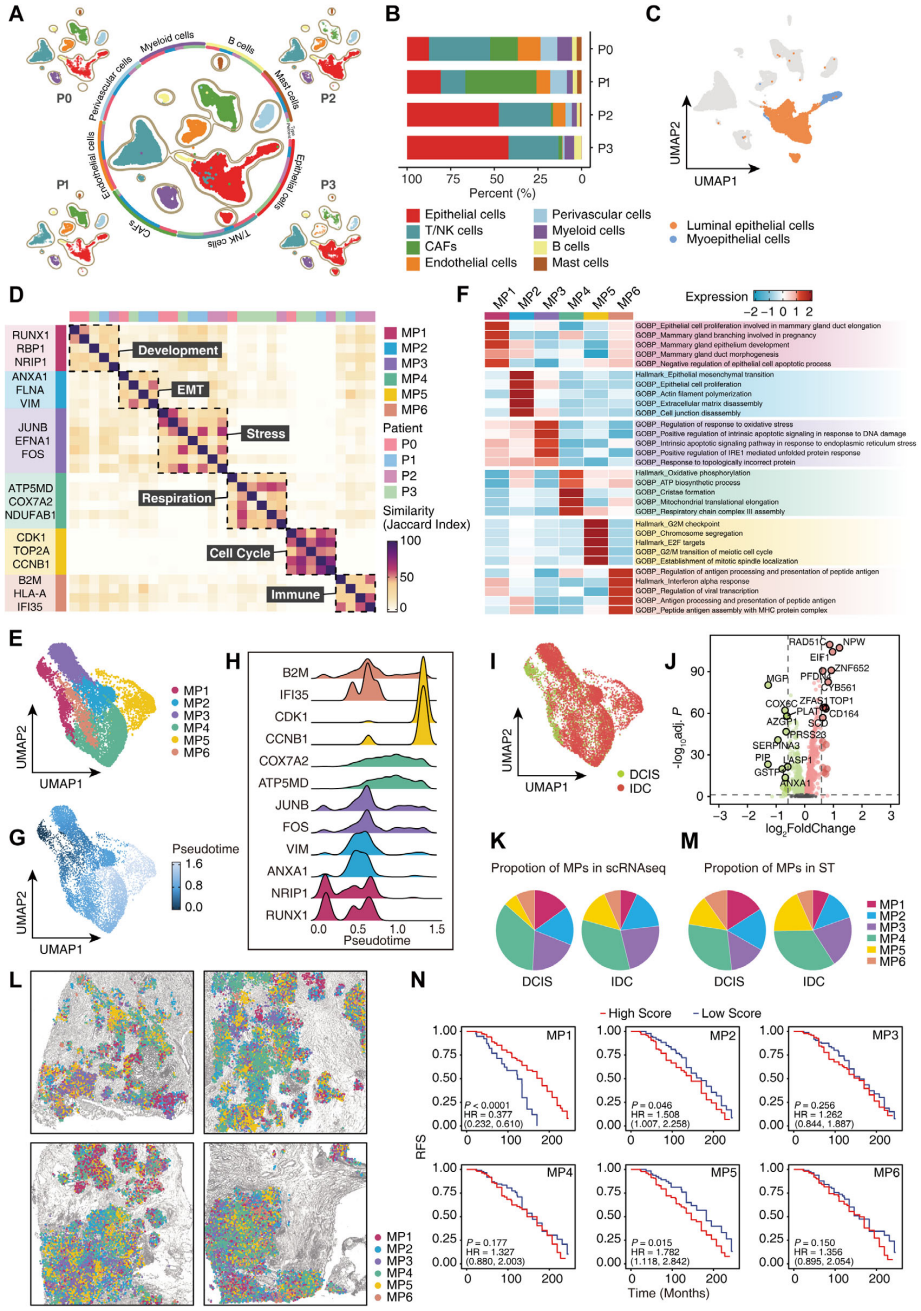
Integrated spatial transcriptomics and scRNAseq identify malignant epithelial meta‐programs distinguishing DCIS from IDC. (A) UMAP visualization of scRNAseq data, with sample‐specific UMAP plots shown around the main embedding. The two surrounding rings denote cell‐type annotations and the relative contribution of each sample. (B) Bar plot showing the proportion of cell types in each sample. (C) UMAP showing the localization of luminal epithelial cells and myoepithelial cells within the global scRNAseq landscape. (D) Heatmap showing shared MPs across all samples. Representative genes for each MP are shown on the left row annotation, and sample distribution is shown on the top annotation. (E) UMAP plot showing malignant epithelial cells categorized by MP classification. (F) Heatmap showing pathway enrichment results of malignant epithelial cells categorized by MP classification using GSVA. (G) Pseudotime trajectory of malignant epithelial cells. (H) Expression dynamics of representative marker genes along the pseudotime trajectory. (I) UMAP plot of malignant epithelial cells categorized into DCIS and IDC based on CelEry annotation. (J) DEGs between DCIS and IDC, with the top 10 genes highlighted by black circles. (K) Pie chart illustrating the proportion of each MP subpopulation within DCIS and IDC in scRNAseq data. (L) Distribution of malignant epithelial cells from MP1 to MP6 derived from CellTrek analysis. (M) Proportions of MP1 to MP6 cells within DCIS and IDC regions in ST data. (N) Kaplan–Meier survival curves showing RFS outcomes for MP‐defined malignant epithelial cell subpopulations in the HTAN database. Significance was assessed using the log‐rank test, HR and 95% CI were estimated using univariable Cox proportional hazards regression.

To further investigate the cellular differences between DCIS and IDC at single‐cell resolution, we performed scRNAseq analysis on the four samples. After stringent quality control to remove low‐quality or dying cells and potential doublets, the scRNAseq data from all four samples showed comparable cell yields and mRNA transcript counts, with an average of 7448 cells per sample and a median UMI count of 5495 per cell (Figure S5A,B). In total, 29 793 high‐quality single cells were retained and categorized into eight major cell lineages using canonical marker genes: epithelial cells (10,763, 36.1%; *EPCAM*, *KRT19*, and *CD24*), T/NK cells (8,059, 27.0%; *PTPRC*, *CD3D*, and *CD3E*), CAFs (4,356, 14.6%; *FAP*, *DCN*, and *LUM*), endothelial cells (2,055, 6.9%; *VWF*, *PECAM1*, and *CD34*), perivascular cells (1,748, 5.9%; *RGS5*, *ACTA2*, and *NOTCH3*), myeloid cells (1,517, 5.1%; *CD68*, *LYZ*, and *HLA‐DRA*), B cells (839, 2.8%; *CD79A*, *MS4A1*, and *IGHG1*) and mast cells (456, 1.5%; *HPGDS*, *TPSB2*, and *RGS13*) (Figure 3A and Figure S5C). Cell type‐specific marker expression was confirmed by Uniform Manifold Approximation and Projection (UMAP) plots (Figure S5D). All major cell types were detected across all four samples, although their relative abundances varied among patients, reflecting inter‐patient cellular diversity (Figure 3B).

Because DCIS predominantly arises from luminal epithelial cells, we next focused on the malignant luminal epithelial compartment. Myoepithelial cells were excluded based on canonical markers, including *KRT5* and *KRT14* (Figure 3C), and epithelial subclones with low CNV burdens were removed as non‐malignant epithelial cells (Figure S6A–D). After these filtering steps, 8866 malignant epithelial cells were extracted from the complete scRNAseq dataset. Molecular subtype analysis using SCSubtype showed that 71.2%–95.4% of malignant epithelial cells across the four samples were classified as Luminal A/B, consistent with the HR‐positive status of all patients. Notably, sample P3 showed a relatively higher proportion of Luminal B cells and Her2E cells, possibly related to its HER2 amplification (Figure S5E).

To characterize the transcriptional states of malignant epithelial cells, we applied nonnegative matrix factorization (NMF) to identify recurrent tumor cell expression programs, each defined by a coherent set of co‐expressed genes within individual tumors. Using previously established methods [23], we identified 31 robust NMF programs across the four samples (Figure S7A). These programs were further grouped into six meta‐programs (MPs) shared across multiple samples by determining the optimal number of clusters (Figure S7B,C). Each MP was defined by a distinct set of genes (Table S2), including mammary gland development (MP1), EMT (MP2), cellular stress response (MP3), cellular respiration (MP4), cell cycle (MP5), and immune response (MP6) (Figure 3D). Notably, the three DCIS‐upregulated genes identified in the matched‐subclone analysis, *MGP*, *PLAT*, and *SERPINA3*, were all among the top‐ranked genes in MP1, indicating a potential association between MP1 and the DCIS state.

We next annotated malignant epithelial cells according to the six MPs at single‐cell resolution. By calculating the Jaccard similarity between the representative genes of each MP and malignant epithelial cell subpopulations (Table S3), epithelial tumor cells were classified into six MP‐associated subgroups (Figure 3E). The distribution of MP scores on the UMAP closely aligned with this classification, supporting the robustness of the MP‐based annotation (Figure S8A). To further define the biological characteristics of each MP, we performed GSVA for each MP‐associated subgroup (Figure 3F). MP1 was enriched in pathways related to mammary gland morphogenesis and development, suggesting a more differentiated transcriptional state with lower malignant potential. MP2 was associated with EMT, extracellular matrix (ECM) organization, and cell adhesion disassembly, consistent with enhanced invasive capacity. MP3 showed enrichment of stress‐ and apoptosis‐related pathways, suggesting adaptation to cellular stress. MP4 was enriched in oxidative phosphorylation, reflecting elevated oxidative metabolic activity. MP5 showed strong enrichment of cell cycle and mitotic pathways, consistent with a highly proliferative phenotype. Finally, MP6 displayed upregulation of immune‐related signaling, implying a role in immunological regulation within malignant epithelial cells. These pathway‐level findings were consistent with the gene‐level annotations of the MPs and were further supported by corresponding pathway scores (Figure S8B). Pseudotime and RNA velocity analyses both placed MP1 at the early stage of the inferred developmental trajectory (Figure 3G and Figure S9A,B). Along the pseudotime axis, malignant epithelial cells showed a transition from high expression of key MP1 genes, including *RUNX1* and *NRIP1*, toward increased expression of genes associated with MP2, MP3, MP4, and MP6, and ultimately toward elevated expression of MP5‐associated genes, including *CDK1* and *CCNB1* (Figure 3H and Figure S9C). Consistently, transcription factor (TF) activity analysis using pySCENIC identified MP‐specific regulatory programs (Figure S9D). In particular, the development‐related TFs *RARB* and *STAT3* were preferentially active in MP1 [24, 25], whereas MP5 showed activation of key cell cycle regulators, including members of the *E2F* family [26].

Given the functional similarity between MPs and DCIS or IDC, we annotated malignant epithelial cells from the scRNAseq data as DCIS or IDC using CelEry based domain prediction [27], which leverages spatial information learned from ST data through a feedforward deep neural network, thereby enabling assessment of the relationship between MPs and pathological types (Figure 3I). Differential expression gene analysis between cells annotated as DCIS and IDC yielded results consistent with those from ST‐based comparisons, supporting the robustness of the CelEry annotation (Figure 3J and Figure 1C). We then quantified the compositional relationship between MPs and DCIS or IDC annotations. This analysis revealed a marked shift in MP composition between the two pathological types: cells assigned to MP1 were predominantly annotated as DCIS, whereas cells assigned to MP5 were more frequently annotated as IDC (Figure 3K). To further validate this spatial association, we jointly embedded scRNAseq and ST data using CellTrek [14]. This analysis confirmed that MP1 cells were mainly localized within DCIS regions, whereas MP5 cells were enriched in IDC regions (Figure 3L,M). Consistently, stratification of malignant epithelial cells into MP score‐defined groups further supported the preferential association of MP1 with DCIS and MP5 with IDC (Figure S10A,B), which was also reflected by the spatial distribution of MP scores across ST spots (Figure S10C).

Based on the close relationship between MP activity and DCIS or IDC pathological types, we next assessed the clinical relevance of the MPs in DCIS progression. Survival analysis showed that a high MP1 score was associated with improved RFS (HR = 0.377, 95% CI: 0.232–0.610), whereas a high MP5 score predicted poorer survival outcomes (HR = 1.782, 95% CI: 1.118–2.842) (Figure 3N). These results support the interpretation that MP1 represents a more differentiated and less aggressive transcriptional state, whereas MP5 is associated with a highly proliferative and clinically unfavorable phenotype, consistent with their respective molecular characteristics. Taken together, these findings suggest that MP1 is preferentially associated with DCIS and reflects a relatively differentiated epithelial state with lower malignant potential, whereas MP5 is more characteristic of IDC and is associated with proliferative and aggressive transcriptional features.

### 2.4 Different Immune‐Tumor Crosstalk Drives Microenvironmental Alterations in DCIS and IDC

**FIGURE 4:**
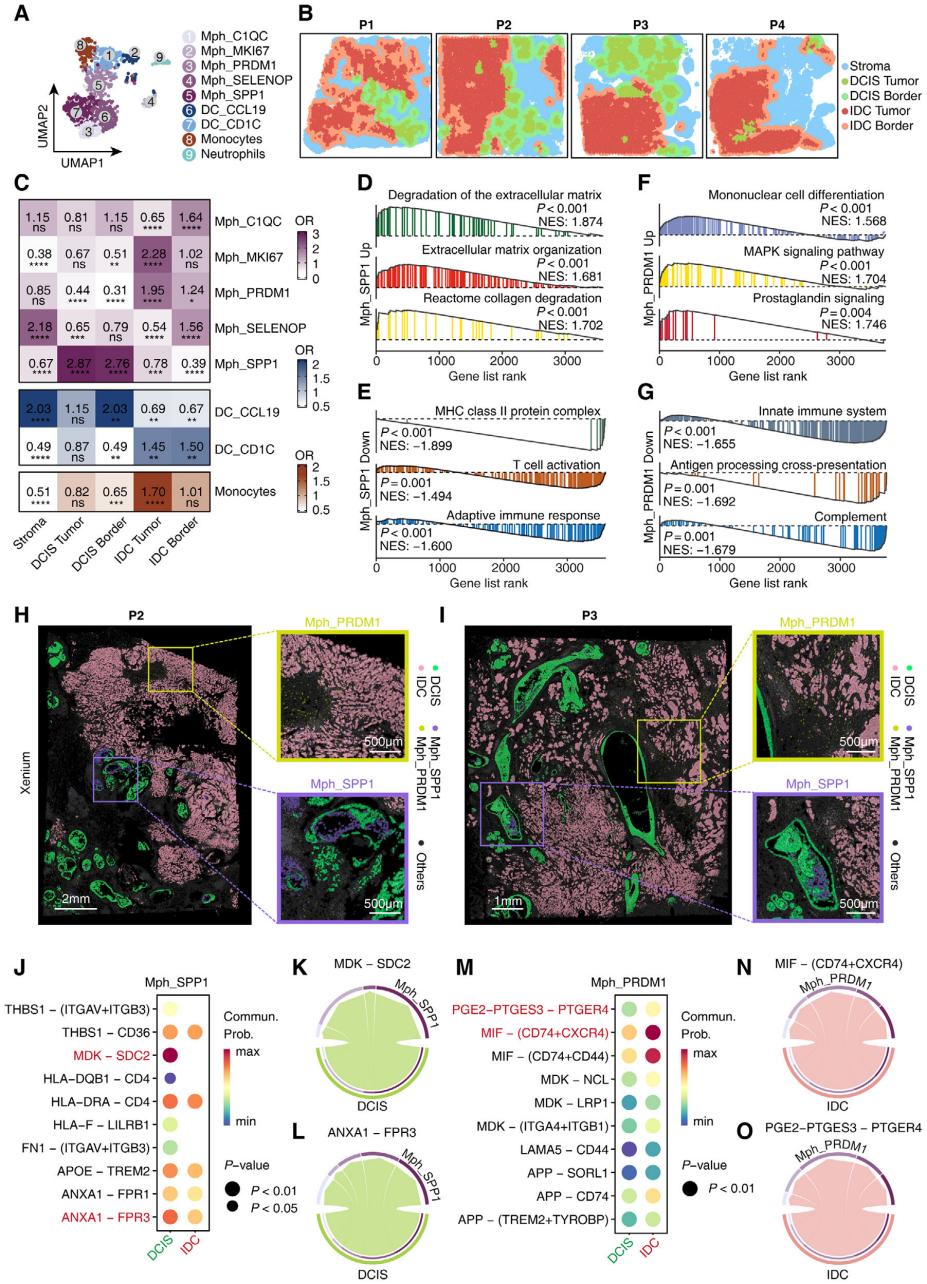
Characterization of myeloid cell subpopulations and their interactions with DCIS and IDC. (A) UMAP plot of myeloid cells, colored by myeloid subpopulations. (B) Spatial mapping of DCIS, IDC, their border area, and the stroma by integrating ST and scRNAseq data using CellTrek. (C) ORs of myeloid cell subpopulations occurring across different tissue regions. Neutrophils were not included in the analysis due to low cell count (< 50 cells). Statistical significance was assessed using Fisher's exact test, with adjustment for multiple comparisons using the BH method; ^****^ *p* < 0.0001, ^***^ *p* < 0.001, ^**^ *p* < 0.01, ^*^ *p* < 0.05, ns, not significant. (D) GSEA enrichment of upregulated pathways for Mph_SPP1. (E) GSEA enrichment of downregulated pathways for Mph_SPP1. (F) GSEA enrichment of upregulated pathways for Mph_PRDM1. (G) GSEA enrichment of downregulated pathways for Mph_PRDM1. (H, I) Xenium spatial transcriptomic visualization of DCIS, IDC, Mph_PRDM1, and Mph_SPP1, with zoomed‐in views of the regions of interest (ROI) for Mph_PRDM1 and Mph_SPP1 shown on the right. (J) Top 10 upregulated ligand‐receptor interactions between DCIS and Mph_SPP1, with dot size representing statistical significance. (K, L) Representative upregulated ligand‐receptor interactions between DCIS and Mph_SPP1, where arrow width indicates interaction strength. (M) Top 10 upregulated ligand‐receptor pairs between IDC and Mph_PRDM1, with dot size representing statistical significance. (N, O) Representative upregulated ligand‐receptor interactions between IDC and Mph_PRDM1, where arrow width indicates interaction strength.

Tumor‐infiltrating immune cells are highly heterogeneous and play critical roles in tumor progression. After filtering out low‐quality cells, we identified 8911 immune cells from the scRNAseq dataset and categorized them into three major immune lineages: T/NK cells, B cells, and myeloid cells. Based on canonical marker genes (Figure S11A–D), we further annotated 9 myeloid subpopulations, 13 T/NK cell subpopulations, and 4 B cell subpopulations (Figure 4A and Figure S12A,B).

To investigate the spatial localization and relative abundance of immune cell subpopulations within the TME, we leveraged scRNAseq data and mapped defined immune cells to their spatial coordinates in the ST dataset using CellTrek. Given that the tumor–stromal boundary represents a biologically active interface that can shape cancer cell behavior during invasion [28], we defined the tumor border as the region extending 200 µm outward from the tumor edge (border area), whereas areas beyond this distance were classified as stroma (Figure 4B). We then calculated odds ratios (ORs) to compare the spatial distribution of immune cell subpopulations across DCIS and IDC regions (Figure 4C and Figure S12C,D). Using an adjusted *p* < 0.01 and an OR > 1.2 or < 0.8 as thresholds, we identified immune subpopulations that were enriched in DCIS but depleted in IDC, and vice versa. After excluding proliferative cell populations, myeloid cells emerged as the most spatially distinct immune subpopulation, with macrophages characterized by *SPP1* expression (Mph_SPP1) preferentially localized in DCIS and macrophages characterized by *PRDM1* expression (Mph_PRDM1) enriched in IDC (Figure 4C). The average cell2location‐inferred frequencies of these two macrophage subpopulations further supported this observation, showing that Mph_SPP1 was more abundant in DCIS than in IDC within both tumor and border regions, whereas Mph_PRDM1 exhibited the opposite pattern (Figure S13A,B). Gene set enrichment analysis (GSEA) revealed that genes upregulated in Mph_SPP1 were significantly enriched in pathways associated with extracellular matrix organization and degradation, suggesting a potential role in basement membrane disruption and facilitation of invasion (Figure 4D,E and Figure S13C). By contrast, Mph_PRDM1 was predominantly enriched in IDC and showed strong activation of immunosuppression‐related pathways, suggesting a potential role in shaping an immunosuppressive microenvironment during IDC progression (Figure 4F,G and Figure S13D). These pro‐progression features were further supported by BayesPrism‐based deconvolution of the HTAN dataset, in which higher inferred proportions of Mph_SPP1 and Mph_PRDM1 were both associated with worse RFS (Mph_SPP1: HR = 1.711, 95% CI: 1.139–2.569; Mph_PRDM1: HR = 1.534, 95% CI: 1.039–2.265) (Figure S13E,F).

We next employed high‐resolution in situ spatial transcriptomics (Xenium) to validate these findings. Deconvolution with Robust Cell Type Decomposition (RCTD) confirmed the spatial distribution patterns of Mph_SPP1 and Mph_PRDM1, with Mph_SPP1 preferentially localized near DCIS regions and Mph_PRDM1 enriched in IDC regions (Figure 4H,I). mIHC further confirmed the preferential enrichment of Mph_SPP1 in DCIS and Mph_PRDM1 in IDC (Figure S13G,H). To explore potential interactions between DCIS, IDC, and their preferentially associated macrophage subpopulations, we used CellChat [29] to infer ligand‐receptor communication. In DCIS, ligand‐receptor analysis suggested that the ANXA1‐FPR3 axis may contribute to Mph_SPP1 recruitment [30], whereas the MDK‐SDC2 pathway may help sustain its matrix‐remodeling and pro‐invasive features (Figure 4J–L) [31]. Together with the enrichment of ECM organization and degradation pathways, these interactions suggest a potential role for Mph_SPP1 in basement membrane remodeling and local invasion. In IDC, the MIF‐(CD74+CXCR4) axis was predicted to facilitate macrophage recruitment [32], whereas activation of the PGE2‐PTGES3‐PTGER4 pathway was associated with an immunosuppressive macrophage phenotype, consistent with the elevated immunosuppressive pathway scores observed in Mph_PRDM1 (Figure 4M–O).

Notably, although no lymphocyte subpopulation showed significant preferential spatial distribution, the exhaustion score of CD8_Tex cells, indicative of an exhausted immune phenotype, was higher in IDC than in DCIS (Figure S12E). Together with the immunosuppressive features of Mph_PRDM1, this finding suggests that Mph_PRDM1 may contribute to the maintenance of CD8 T cell exhaustion, as supported by the inferred MIF‐associated communication between Mph_PRDM1 and CD8_Tex cells [33] (Figure S13I). Among B cells, mature IgG plasma cells (B_IgG_mature) were more abundant in IDC than in DCIS (Figure S12D). For B‐cell receptor (BCR) clonotype analysis, after excluding P1 because of low T/B cell infiltration (Figure S14A), we observed greater B‐cell clonal diversity in IDC than in DCIS regions. Moreover, a clear clonal association between tertiary lymphoid structures (TLSs) and IDC, but not DCIS, was observed across three samples (Figure S14B–D). Taken together, these findings suggest that immune remodeling during DCIS‐to‐IDC progression involves distinct macrophage associated programs, with DCIS characterized by Mph_SPP1 related interactions that may facilitate basement membrane remodeling and early invasion, whereas IDC was marked by Mph_PRDM1 associated immunosuppression and CD8 T cell exhaustion, along with increased B cell clonal diversity.

### 2.5 Distinct CAF Subpopulations Exhibit Lesion‐Dependent Roles in DCIS and IDC

**FIGURE 5:**
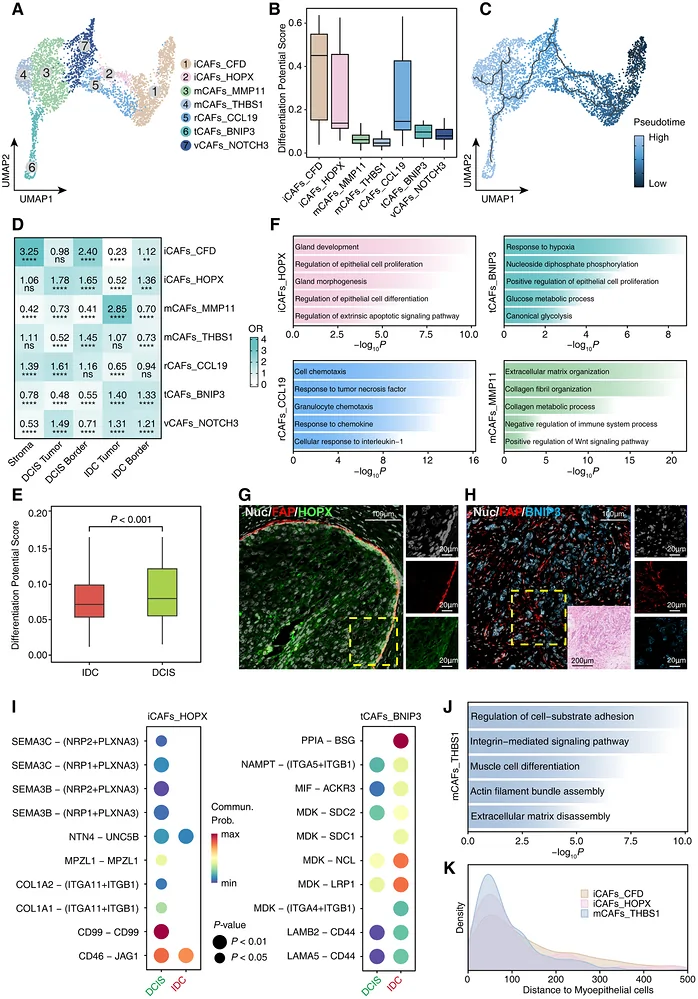
Characterization of CAF subpopulations and their interactions with DCIS and IDC. (A) UMAP plot of CAFs, colored by CAF subpopulations. iCAFs, inflammatory CAFs; mCAFs, matrix CAFs; rCAFs, reticular‐like CAFs; tCAFs, tumor‐like CAFs; vCAFs, vascular CAFs. (B) Boxplot showing differentiation potential scores of CAF subpopulations calculated using CytoTRACE2. (C) Pseudotime trajectory of CAFs, with cells colored by pseudotime and the trajectory line shown. (D) ORs for CAF subpopulations enrichment across different tissue regions. Statistical significance was assessed using Fisher's exact test, with adjustment for multiple comparisons using the BH method; ^****^ *p* < 0.0001, ^***^ *p* < 0.001, ^**^ *p* < 0.01, ns, not significant. (E) Boxplot showing differential potential scores of CAFs in DCIS and IDC. Statistical significance was assessed using a two‐sided Wilcoxon test. (F) GO pathway enrichment analysis for iCAFs_HOPX, rCAFs_CCL19, tCAFs_BNIP3, and mCAFs_MMP11. (G) Representative mIHC staining images of FAP and HOPX in DCIS. (H) Representative mIHC staining images of FAP and BNIP3 in IDC. The inset shows the corresponding H&E image. (I) Top 10 upregulated ligand‐receptor pairs between DCIS and iCAFs_HOPX and between IDC and tCAFs_BNIP3. Dot size represents statistical significance. (J) GO pathway enrichment analysis for mCAFs_THBS1. (K) Density distribution of iCAFs_CFD, iCAFs_HOPX, and mCAFs_THBS1 according to their distance to myoepithelial cells.

Given the critical role of stromal cells in maintaining the tumor microenvironment and influencing tumor invasion, we next focused on the relationship between distinct stromal subpopulations and DCIS or IDC. After a second round of filtering, 4222 CAFs, 2030 endothelial cells, and 1760 perivascular cells from the scRNAseq dataset were reclassified into several subpopulations based on marker gene expression profiles (Figure 5A and Figures S15A–C and S16A,B). Among these subpopulations, CAFs showed the highest cellular abundance and greatest heterogeneity. Differentiation potential scores calculated using CytoTRACE2 revealed substantial heterogeneity among stromal subpopulations (Figure 5B) [34], which was further supported by pseudotime trajectory analysis using Monocle3 (Figure 5C). Spatial analysis revealed distinct localization patterns among CAF subpopulations (Figure 5D), which were corroborated by average cell2location inferred frequencies and spatial mapping (Figure S17A). Applying the same regional definitions and filtering criteria used for the immune cell analysis, we found that iCAFs_HOPX and rCAFs_CCL19 were preferentially enriched in DCIS tumor regions, whereas tCAFs_BNIP3 and mCAFs_MMP11 were significantly enriched in IDC tumor regions. Notably, DCIS enriched CAF subpopulations, including iCAFs_HOPX and rCAFs_CCL19, exhibited higher differentiation potential scores than IDC enriched subpopulations, including tCAFs_BNIP3 and mCAFs_MMP11, suggesting that CAF states become progressively specialized during DCIS‐to‐IDC transition, potentially reflecting remodeling by the IDC microenvironment (Figure 5B,D). This pattern was further supported by ST data, in which CAFs in DCIS regions displayed significantly higher differentiation potential scores than those in IDC regions (Figure 5E).

Functional annotation further revealed distinct biological features among CAF subpopulations. The iCAFs_HOPX population was significantly associated with epithelial development and proliferation‐related pathways, suggesting a potential role in maintaining a development‐associated epithelial niche in DCIS. In parallel, rCAFs_CCL19 was enriched in pathways related to myeloid cell migration and chemotaxis, implicating a potential role in immune cell recruitment. In IDC, tCAFs_BNIP3 was predominantly associated with hypoxia and glycolysis pathways, processes linked to poor prognosis [35], whereas mCAFs_MMP11 was associated with ECM remodeling and immunosuppressive pathways, highlighting its potential contribution to an invasive and immunosuppressive stromal microenvironment in IDC (Figure 5F).

To extend these observations, we performed BayesPrism‐based deconvolution of HTAN bulk RNAseq data, which revealed the prognostic relevance of the identified CAF subpopulations. A higher inferred proportion of iCAFs_HOPX was associated with favorable outcomes (HR = 0.443, 95% CI: 0.266–0.738), supporting a potential role for this CAF subpopulation in maintaining epithelial developmental programs and a less aggressive stromal state. In contrast, a higher inferred proportion of tCAFs_BNIP3 was linked to poorer prognosis (HR = 1.779, 95% CI: 1.177–2.690), consistent with its metabolic stress features and enrichment in IDC (Figure S17B).

We further examined the spatial patterns of these CAF subpopulations using Xenium (Figure S17C) and mIHC staining (Figure 5G,H), both of which confirmed the preferential localization of iCAFs_HOPX in DCIS and tCAFs_BNIP3 in IDC. CellChat analysis revealed differentially activated ligand receptor interactions between DCIS and IDC. In iCAFs_HOPX, CD99‐CD99 signaling was more strongly activated in DCIS than in IDC, suggesting that this interaction may contribute to the adhesion, differentiation, and apoptosis regulation of DCIS [36]. By contrast, in IDC, tCAFs_BNIP3 showed activation of pathways associated with CAF functional maturation, including PPIA‐BSG signaling, which is linked to metabolic reprogramming [37], and MDK driven signaling, which has been implicated in CAF proliferation [38] (Figure 5I).

Notably, mCAFs_THBS1, which expressed ECM, cell adhesion, and myofibroblast‐related signatures (Figure 5J), was predominantly detected in the DCIS border region (Figure 5D and Figure S17D). Prognostic analysis showed that a higher inferred proportion of mCAFs_THBS1 was associated with worse RFS in DCIS patients (Figure S17E), indicating a potential pro‐invasive role. Considering that myoepithelial cells are located along the DCIS border and play an important role in restricting DCIS invasion, we next examined whether mCAFs_THBS1 may influence myoepithelial cell function. Cell distance analysis based on CellTrek data showed that mCAFs_THBS1 exhibited the strongest spatial proximity to myoepithelial cells among CAF subpopulations enriched in the DCIS border region (Figure 5K). Ligand receptor analysis further suggested that mCAFs_THBS1 may perturb myoepithelial barrier function through laminin or collagen integrin signaling, potentially contributing to basement membrane remodeling and tumor invasion (Figure S17F) [39, 40].

Within the stromal compartment, we further examined the distributional patterns of endothelial and perivascular subpopulations across DCIS and IDC. Among these subpopulations, arterial endothelial cells were preferentially enriched in IDC (Figure S16C); however, their relative abundance was not significantly associated with RFS in patients with DCIS (Figure S16D). Taken together, these findings identify CAFs as the stromal populations most closely associated with DCIS progression, among which iCAFs_HOPX may restrain progression by maintaining epithelial differentiation, whereas tCAFs_BNIP3 and mCAFs_THBS1 may promote progression through metabolic reprogramming and myoepithelial dysfunction, respectively.

### 2.6 Development of a Machine Learning–Based Model for High‐Risk DCIS Prediction

**FIGURE 6:**
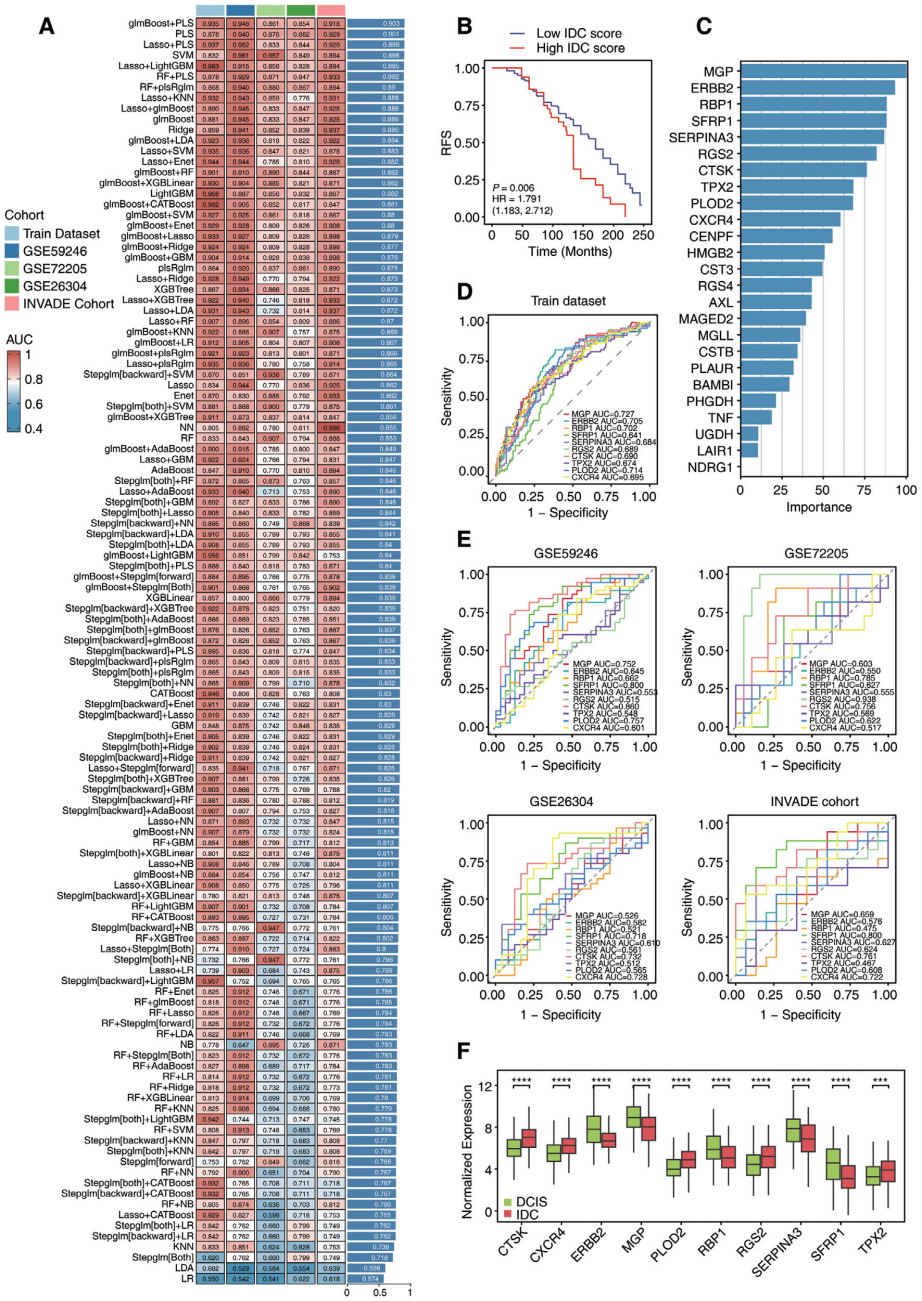
Distinct signatures between DCIS and IDC predict the invasive‐like score of DCIS. (A) Heatmap of AUC performance for 119 machine‐learning models in the training and validation sets; glmBoost+PLS achieved the highest mean AUC of 0.903. (B) Kaplan–Meier survival curves showing RFS outcomes for DCIS cases with high and low invasive‐like scores in the HTAN database. Significance was assessed using the log‐rank test. HR and 95% CI were estimated using univariable Cox proportional hazards regression. (C) Gene importance in the glmBoost+PLS model. (D, E) ROC curves depicting the predictive performance of the top 10 important genes across the training (D) and validation (E) cohorts. (F) Box plots comparing the expression of the top 10 important genes between DCIS and IDC in training and validation datasets. Statistical significance was assessed using a two‐sided Wilcoxon test. ^****^ *p* < 0.0001, ^***^ *p* < 0.001.

It has been proposed that DCIS lesions with invasive‐like properties may have a higher likelihood of progression [41]. To assess the clinical relevance of the cell subpopulations identified in our study and establish a model for predicting DCIS progression risk, we developed an IDC‐like scoring model based on multi‐omics derived features. Specifically, we extracted the 100 genes defining each of MP1 and MP5 (Table S2), together with the top 100 differentially expressed genes from cell populations that showed preferential enrichment in DCIS or IDC and significant prognostic relevance (Table S4), including Mph_SPP1, Mph_PRDM1, iCAFs_HOPX, and tCAFs_BNIP3. After removing basal and normal‐like samples and performing grid search based hyperparameter tuning, we evaluated 119 machine learning models across the training cohort and four external validation cohorts. A heatmap of AUC performance identified the glmBoost+PLS model as the best‐performing classifier, with the highest mean AUC of 0.903 (Figure 6A). This result indicates that the glmBoost+PLS model robustly distinguishes DCIS from IDC, with a higher prediction score representing a more IDC‐like transcriptional state. We then applied this model to the HTAN dataset. Kaplan‐Meier analysis showed that samples with high IDC‐like scores had significantly worse RFS (HR = 1.791, 95% CI: 1.183–2.713), suggesting that the IDC‐like score captures clinically relevant progression or recurrence risk (Figure 6B).

We next investigated which genes contributed most strongly to the predictive performance of this model. Overall, 25 genes derived from our datasets were retained in the final model (Figure 6C). Based on their feature importance rankings, the top 10 genes were selected as representative model genes for subsequent biological analyses. Importantly, most of these genes showed robust predictive performance across the training and validation datasets (Figure 6D,E). Box plot analysis revealed distinct expression patterns between DCIS and IDC. *ERBB2*, *MGP*, *RBP1*, *SERPINA3*, and *SFRP1* were significantly elevated in DCIS, whereas *CTSK*, *CXCR4*, *PLOD2*, *RGS2*, and *TPX2* were significantly elevated in IDC (Figure 6F), suggesting that these genes may contribute to IDC‐like prediction in opposite directions.

**FIGURE 7:**
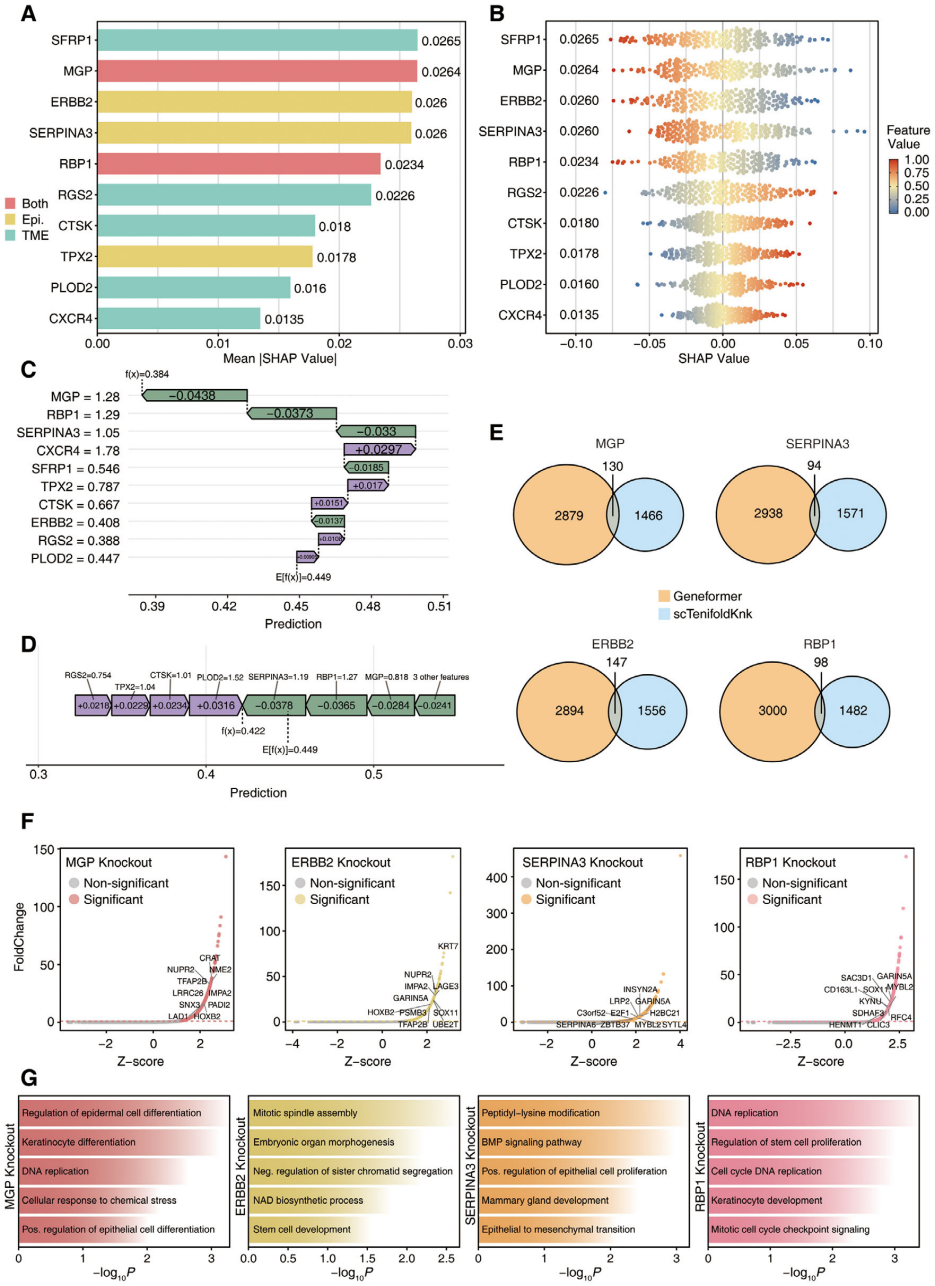
SHAP interpretation of the glmBoost+PLS model. (A) Bar plot showing the mean absolute SHAP values of the top 10 important genes in the glmBoost+PLS model. (B) Beeswarm plot showing the distribution of SHAP values for the top 10 important genes in the glmBoost+PLS model. (C) Waterfall plot illustrating the cumulative feature contributions of the top 10 important genes to the model prediction output of the glmBoost+PLS model. (D) Force plot showing the positive and negative contributions of the top 10 important genes to the prediction output of the glmBoost+PLS model. (E) Venn diagram indicates the overlap of perturbed genes identified by scTenifoldKnk and Geneformer after virtual knockout of *MGP*, *ERBB2*, *SERPINA3*, and *RBP1* in scRNAseq data from the HTAN pure DCIS cohort. (F) Dot plot showing the magnitude of gene changes after virtual knockout of *MGP*, *ERBB2*, *SERPINA3*, and *RBP1*, highlighting the top 10 shared perturbed genes identified by both scTenifoldKnk and Geneformer in scRNAseq data from the HTAN pure DCIS cohort. (G) GO pathway enrichment analysis of significantly perturbed genes shared by scTenifoldKnk and Geneformer following virtual knockout of *MGP*, *ERBB2*, *SERPINA3*, and *RBP1* in scRNAseq data from the HTAN pure DCIS cohort.

To further interpret the model, we performed SHAP analysis on the glmBoost+PLS classifier to quantify the contribution of each feature to DCIS and IDC classification. The SHAP bar plot showed that the mean absolute SHAP values of the top 10 genes ranged from 0.0265 to 0.0135 (Figure 7A). The SHAP beeswarm plot further illustrated the directionality of feature effects. Consistent with their expression patterns, higher expression of *ERBB2*, *MGP*, *RBP1*, *SERPINA3*, and *SFRP1* was associated with negative SHAP values, indicating a shift toward DCIS‐like prediction. In contrast, higher expression of *CTSK*, *CXCR4*, *PLOD2*, *RGS2*, and *TPX2* was associated with positive SHAP values, indicating a shift toward IDC‐like prediction (Figure 7B). A representative SHAP waterfall plot showed the cumulative contribution of individual genes to the final prediction, with the model output changing from the base value E[f(x)] = 0.449 to f(x) = 0.384. Consistent with the average SHAP patterns, *MGP*, *RBP1*, *SERPINA3*, and *ERBB2* contributed negatively to the prediction, whereas *CXCR4*, *TPX2*, *CTSK*, *RGS2*, and *PLOD2* contributed positively (Figure 7C). Similar feature effects were also observed in the force plot (Figure 7D).

Among these genes, *MGP*, *ERBB2*, *SERPINA3*, and *RBP1* were derived from representative genes of MP1, whereas *TPX2* was derived from the proliferative MP5 program, consistent with their opposite effects on IDC‐like prediction. To further explore their biological functions, we performed virtual knockout analysis of these genes in pure DCIS scRNAseq datasets from HTAN. By integrating results from Geneformer and scTenifoldKnk, we identified 130, 147, 94, 98, and 93 shared perturbed genes for the selected genes, respectively (Figure 7E,F and Figure S18A,B). GO enrichment analysis showed that virtual deletion of these genes primarily affected pathways related to epithelial differentiation and cell cycle regulation (Figure 7G and Figure S18C), consistent with the functional annotations of MP1 and MP5. These results suggest that the model genes are not merely predictive markers but may functionally contribute to DCIS‐to‐IDC progression by regulating epithelial differentiation and cell cycle related processes.

In summary, we developed a machine learning‐based IDC‐like scoring model with strong performance in distinguishing DCIS from IDC and assessing DCIS progression risk. This model further supports our multi‐omics finding that distinct epithelial gene expression programs and preferential TME distribution patterns may shape the progression potential of DCIS.

## 3 Discussion

The coexistence of DCIS and IDC within the same tumor provides a unique opportunity for comprehensive transcriptomic and genomic characterization to elucidate the molecular differences and evolutionary relationship between these two components. Leveraging the power of single‐cell omics and spatial transcriptomics to interrogate both malignant and TME cells at high resolution, our study presents an integrated transcriptomic and genomic landscape across tumor and stromal compartments. We identified distinct marker genes, specific gene expression modules, and their correlations with clinical outcomes. We also characterized how the TME shapes the divergent biological features of DCIS and IDC and uncovered spatially enriched cell populations that may promote the acquisition of invasive traits during DCIS progression. Furthermore, we developed a machine learning model based on transcriptional differences between DCIS and IDC, demonstrating its potential to identify high‐risk DCIS while highlighting key genes that may drive the transition from DCIS to invasive disease.

By retaining tissue architecture, spatial transcriptomics enables direct comparison of transcriptional features across coexisting pathological regions representing different stages of disease progression within the same tissue section [42]. Spatial transcriptomic analysis revealed distinct transcriptional programs between coexisting DCIS and IDC regions, with differential expression of representative genes including *MGP* and *SCGB2A2* and enrichment of distinct biological pathways. Furthermore, previous scDNAseq studies have indicated that additional CNV events may emerge during the transition from DCIS to IDC [6, 43], contributing to transcriptional reprogramming during tumor evolution [44]. By integrating ST inferred CNV profiles with scCUTseq data, we reconstructed phylogenetic relationships between matched DCIS and IDC subclones, and identified invasion‐associated transcriptional alterations, including higher expression of *MGP* in DCIS, as well as *PLAT* and *SERPINA3*. Notably, the higher expression of *MGP* and *PLAT* in DCIS was consistent with a recent three‐dimensional study of DCIS progression, which associated these genes with a less invasive state through the maintenance of extracellular matrix homeostasis and epithelial integrity [45]. Similarly, the higher expression of *SERPINA3* in DCIS was consistent with previous studies linking its low expression to poor prognosis in breast cancer [46], suggesting that loss of *SERPINA3* may accompany or contribute to the transition from DCIS to IDC. By contrast, *SCGB2A2*, which was enriched in IDC, has been implicated in enhancing breast cancer cell migration and invasion [47], consistent with the acquisition of a more invasive phenotype.

Unlike distinct cell types, cancer cell states often exist along a continuum, making categorical definition challenging [48]. Our analysis underscores the complexity and substantial heterogeneity in malignant epithelial cell states, largely driven by overlapping gene module expression. Using NMF on scRNAseq data, we identified six robust MPs within malignant epithelial cells. MP1, strongly linked to DCIS, was enriched in pathways involving mammary gland morphogenesis and development, suggesting a more differentiated transcriptional state with lower malignant potential. This aligns with previous findings that DCIS lesions appear to retain epithelial development‐related programs, whereas progression toward IDC is associated with their gradual loss or remodeling [49]. In contrast, MP5, associated with IDC, reflected the high proliferative activity characteristic of invasive tumors. These findings further support differential pathway enrichment observed between DCIS and IDC [50].

The TME is a key component shaping tumor evolution during the transition from DCIS to IDC [51]. The integration of scRNAseq, ST, and Xenium technologies allowed us to investigate spatial TME features of coexisting DCIS and IDC at single‐cell resolution. Macrophage subpopulations have been implicated in promoting invasion by degrading the basement membrane and disrupting cell junctions [52, 53]. In our dataset, Mph_SPP1 macrophages were significantly enriched in DCIS, exhibited elevated activity of ECM degradation pathways, and were associated with poor prognosis in the DCIS cohort, suggesting a potential pro‐invasive function. Given that high‐risk DCIS can harbor hypoxic and centrally necrotic intraductal microenvironments [54], the preferential enrichment and matrix remodeling features of Mph_SPP1 are consistent with previous observations that SPP1^+^ macrophages accumulate in hypoxic and necrotic tumor regions and may facilitate invasion through ECM remodeling processes [55, 56]. In contrast, the IDC microenvironment exhibited a more immunosuppressive phenotype that may promote immune evasion [57], consistent with the enrichment of Mph_PRDM1 macrophages in IDC and their high immunosuppressive scores. IDC regions were also characterized by higher CD8_Tex exhaustion scores and greater B‐cell clonal diversity than DCIS. The increased B‐cell clonal diversity may reflect a more diverse BCR repertoire and broader antigenic stimulation in IDC [58], suggesting persistent tumor antigen exposure. Together with the inferred communication between Mph_PRDM1 macrophages and CD8_Tex cells, these findings suggest that persistent antigen exposure [59], and Mph_PRDM1 associated immunosuppression may jointly contribute to CD8 T lymphocytes dysfunction within the IDC microenvironment.

CAFs can be reprogrammed from normal fibroblasts under the influence of tumor‐derived signals and acquire pro‐tumorigenic functions [60]. In our dataset, CAFs enriched in IDC exhibited a higher degree of differentiation than those in DCIS, reflecting phenotypic shifts in response to the invasive microenvironment. This functional transition may reflect the dynamic reprogramming of fibroblasts by evolving tumor‐derived cues. DCIS enriched CAF subpopulations, such as iCAFs_HOPX, were enriched in epithelial development and gland morphogenesis pathways, suggesting that they may retain homeostatic stromal functions that support epithelial differentiation and tissue organization, thereby restraining invasion. This interpretation is consistent with previous studies showing that fibroblasts can preserve epithelial differentiation and contribute to the control of tumor growth [61]. In the border area surrounding DCIS lesions, mCAFs_THBS1 colocalized with myoepithelial cells and were associated with poor prognosis in patients with DCIS. Given the critical role of myoepithelial cells in maintaining the ductal barrier [62], the inferred communication between mCAFs_THBS1 and myoepithelial cells suggests that this CAF population may impair myoepithelial function and facilitate local invasion. With progression to IDC, sustained exposure to tumor‐derived inflammatory, growth, and metabolic signals may promote further fibroblast reprogramming [63, 64]. Consistent with this possibility, IDC‐enriched tCAFs_BNIP3 were associated with metabolic pathways and may thereby contribute to progression of malignant epithelial cells [65]. Collectively, these findings suggest that CAF functions change dynamically with pathological state and tissue context, shifting from relatively homeostatic states in DCIS toward barrier‐disruptive and metabolically supportive states during the transition to IDC.

The progression risk of DCIS can be assessed by its similarity to IDC, with more IDC‐like lesions considered more likely to progress [19, 41]. Accordingly, we integrated multiple public transcriptomic cohorts and evaluated 119 machine learning model combinations, among which glmBoost+PLS achieved a mean AUC of 0.903 across the training and validation cohorts. Higher IDC‐like scores were associated with worse RFS in the HTAN DCIS cohort, supporting the potential clinical relevance of the model. SHAP analysis showed that *MGP* and *SERPINA3*, identified by matched‐subclone analysis and represented in MP1, contributed negatively to IDC‐like prediction, consistent with our multi‐omics findings. The MP1‐associated genes *ERBB2* and *RBP1* also contributed negatively, whereas the proliferative MP5 gene *TPX2* contributed positively. *RBP1*, which encodes retinol‐binding protein 1, has been reported to promote retinoic acid receptor activity and acinar differentiation in breast epithelial cells while suppressing tumorigenicity [66]. In contrast, *TPX2*, a key regulator of mitotic spindle formation and microtubule nucleation, has been shown to promote breast cancer cell proliferation, migration, and invasion [67]. *ERBB2* showed a negative contribution to IDC‐like prediction in our model, which may appear paradoxical given its well‐established oncogenic role in breast cancer. Indeed, previous studies have shown that *ERBB2* amplification is more frequent in DCIS than in invasive breast cancer and may preferentially promote proliferation rather than invasion [68]. *ERBB2*‐overexpressing DCIS has also been associated more frequently with in situ recurrence than with invasive recurrence [69]. These observations raise the possibility that *ERBB2* amplification arises early during tumor evolution and is subsequently subject to clonal selection, with *ERBB2*‐amplified clones preferentially retained in the in situ component rather than selected during invasion. Nevertheless, this interpretation requires further investigation.

While our study highlights the roles of genomic alteration, malignant epithelial programs, and spatial remodeling of the tumor microenvironment in DCIS‐to‐IDC progression, several limitations should be acknowledged. First, the sample size was relatively small, which may have limited our ability to capture the full extent of microenvironmental heterogeneity in DCIS and IDC. To mitigate this limitation, we integrated multiple large public bulk RNAseq cohorts to assess the robustness and clinical relevance of our findings. Given the limited number of cases in our cohort, the study was not powered to systematically evaluate molecular subtype‐specific patterns, potentially obscuring subtype‐dependent cellular and microenvironmental features. Larger cohorts with adequate representation of each subtype are therefore needed to validate and extend these findings. Second, our single‐cell and spatial analyses were performed on coexisting DCIS and IDC samples. Thus, the DCIS component in these samples may not fully represent pure DCIS, as the tumors in these patients have already demonstrated invasive potential. Further validation in larger cohorts of coexisting DCIS and IDC, as well as additional studies of pure DCIS, will therefore be important to establish the broader relevance of our findings. Third, current technical constraints prevented direct CNV profiling at single‐cell resolution while preserving spatial context. We therefore integrated ST‐inferred CNV profiles with scCUTseq data, thereby providing complementary spatial and single‐cell genomic information.

In conclusion, by integrating ST, scRNAseq, scDNAseq from coexisting DCIS and IDC samples, we systematically characterized the genomic evolution, malignant epithelial programs, and spatial remodeling of the TME associated with DCIS‐to‐IDC progression. We further developed an IDC‐like scoring model that linked these multi‐omics features to DCIS progression risk. Together, our findings provide a multidimensional framework for understanding the biological transition from in situ to invasive disease and may inform future molecular risk assessment and therapeutic investigation in DCIS.

## 4 Methods

### 4.1 Experimental Methods

#### 4.1.1 Breast Cancer Samples

We prospectively collected five fresh breast cancer tissue samples (ID: P0‐P4) from female patients aged 35–56 who were diagnosed and underwent surgery at Qilu Hospital of Shandong University. The main clinicopathological characteristics of the patients are summarized in Table S1. All specimens were obtained from treatment‐naive patients and pathologically confirmed to harbor both DCIS and IDC components. For each sample, a tissue block of approximately 1 × 1 × 1cm^3^ was obtained and evenly divided into three parts. One portion was immersed in sterile tissue storage and transportation medium (Life Science Production, Cat. No. TSTORE‐A) and maintained at +4°C until further processing for scRNAseq. The second portion was embedded in Tissue‐Tek OCT compound (SAKURA, Cat. No. 4583), snap‐frosted in isopentane, and stored at –80°C for subsequent single cell DNA sequencing and spatial transcriptomic profiling. The third portion was fixed in formalin and embedded in paraffin wax for Xenium analysis. The collection and use of all breast tissue samples were approved by the Ethics Committee of Qilu Hospital of Shandong University (Approval No. KYLL‐202107‐152‐4), and written informed consent was obtained from all participating patients.

#### 4.1.2 Single‐Cell RNA Sequencing (scRNAseq)

To prepare the single‐cell suspension, fresh tissue samples were briefly rinsed with PBS and minced into approximately 1mm^3^ fragments on ice using surgical scalpels. The tissue fragments were then enzymatically digested at 37°C for 1 h with gentle shaking in the RPMI 1640‐based digestion medium supplemented with 2 mg/mL collagenase II (Sigma–Aldrich, Cat. No. V900892) and 10 µg/mL DNase I (Sigma–Aldrich, Cat. No. DN25‐100MG). Following digestion, the cell suspension was filtered through a 40 µm cell strainer (Corning, Cat. No. 352340) to remove undigested debris, and the filtrate was centrifuged to collect the cells. To eliminate residual erythrocytes, the cell pellet was resuspended in red blood cell lysis buffer (Solarbio, Cat. No. R1010) and incubated for 3 min at room temperature. The cells were then washed three times with PBS and resuspended in 0.04% BSA in PBS on ice. Finally, total cell number and viability were assessed using an automatic cell counter before downstream applications.

The single‐cell suspension was adjusted to a concentration of 300–600 cells/µL and loaded into a well of a Chromium Next GEM Chip (10x Genomics, Cat. No. 1000120) for processing on the Chromium X Single Cell Gene Expression platform (10x Genomics, Pleasanton, CA, USA). Single‐cell transcriptomic libraries were generated using the Chromium Next GEM Single Cell 3ʹ kit v3.1 (10x Genomics, Cat. No. 1000269), following the manufacturer's protocol. Library quality was assessed using the Agilent 4200 TapeStation System (Agilent Technologies, Santa Clara, CA, USA). Sequencing was performed on the Illumina NovaSeq 6000 platform (Illumina, San Diego, CA, USA) in 150 bp paired‐end mode, aiming for an average depth of 0.02 million (M) reads per cell.

#### 4.1.3 Single‐Cell DNA Sequencing With scCUTseq

For the preparation of single‐nucleus suspension, we followed the Tapestri Frozen Tissue Nuclei Extraction Protocol (https://missionbio.com/resources/user‐guides/), as previously described [21]. Briefly, OCT‐embedded frozen tissues were sectioned into 70 µm‐thick slices and minced into small fragments on dry ice. The tissue fragments were then transferred into the tissue lysis buffer and digested for 15 min with gentle rotation. Lysis was terminated by adding stop solution, followed by filtration and centrifugation to isolate the nuclei. The nuclei were subsequently fixed with a methanol/acetic acid solution and resuspended in 1×PBS containing 5 mM EDTA and 0.05% NaN3 for long‐term storage. Before sorting, the nuclear suspension was filtered through a 20 µm cell strainer (Sysmex, Cat. No. 04‐004‐2315), stained with Hoechst 33342 (Thermo Fisher Scientific, Cat. No. 62249) and stored at +4°C.

Before sorting, each well of a 384‐well plate was prefilled with 5 µL of mineral oil. Single nuclei were sorted using a BD Aria III cell sorter equipped with a 100 µm nozzle (BD Biosciences, San Jose, CA, USA). After sorting, the plates were immediately sealed, centrifuged, and stored at ‐20°C for subsequent processing. Each nucleus was lysed and subjected to whole‐genome amplification using the MALBAC kit (Yikon Genomics, Cat. No. YK001A) in a miniaturized reaction volume enabled by the I.DOT nanodispensing system (DISPENDIX GmbH, Stuttgart, Germany). The amplified DNA was digested with NlaIII restriction enzyme (NEB, Cat. No. R0125) and ligated to the scCUTseq adapters (homemade as previously described [21]). All samples from each well were then carefully pooled, and the mineral oil overlay was thoroughly removed. The pooled DNA was purified using VAHTS DNA Clean Beads (Vazyme, Cat. No. N411) and then fragmented with a Bioruptor Pico sonication device (Diagenode, Denville, NJ, USA), targeting a peak fragment size of ∼200 base pairs (bp). The sheared DNA was then processed through in vitro transcription (IVT; Thermo Fisher Scientific, Cat. No. AM1333), RA3 adapter ligation (NEB, Cat. No. M0242L), reverse transcription (Thermo Fisher, Cat. No. 18090050), and final library amplification (NEB, Cat. No. M0544). The resulting libraries were quality‐checked using the Qsep 100 analyzer (BiOptic, New Taipei City, Taiwan) and sequenced on an Illumina NovaSeq 6000 platform (Illumina, San Diego, CA, USA) in 150 bp paired‐end mode, targeting an average depth of 1 m reads per nucleus.

#### 4.1.4 Spatial Transcriptomic Profiling With Visium

Spatial transcriptomic amplification and library preparation were conducted by Beijing Bioao Biotechnology (Beijing, China) using the Visium Spatial Gene Expression platform (10x Genomics, Pleasanton, CA, USA), following the manufacturer's protocols. In brief, OCT‐embedded frozen tissue samples were cryosectioned into 10 µm‐thick slices using a cryostat and carefully mounted within the capture areas of Visium Spatial slides (10x Genomics, Cat. No. PN‐1000184). After fixation, the sections were stained with hematoxylin and eosin (H&E) to obtain high‐resolution morphological imaging. Subsequent tissue permeabilization allowed mRNA to be captured by spatially barcoded oligonucleotides. Reverse transcription, second‐strand synthesis, cDNA amplification, and library preparation were performed according to the manufacturer's instructions. The final libraries were sequenced on an Illumina NovaSeq 6000 platform using a paired‐end 150 bp strategy targeting an average depth of 0.05 m reads per tissue spot. Approximately 4000 tissue spots per section were captured and included in downstream analyses.

#### 4.1.5 In Situ Gene Expression Profiling With Xenium

In situ gene expression profiling was performed by Fynn Biotechnologies (Jinan, China) following the Xenium In Situ Gene Expression Workflow (10x Genomics, Pleasanton, CA, USA). Formalin‐fixed, paraffin‐embedded (FFPE) tissue samples were sectioned into 5 µm‐thick slices and mounted onto Xenium slides (10x Genomics, Cat. No. PN‐1000460). The sections underwent a series of preparatory steps, including deparaffinization with xylene, rehydration through a graded ethanol series, and decrosslinking. After tissue permeabilization, Xenium circularizable DNA probes (Human Breast Gene Expression Panel, 280 genes) were hybridized to their complementary mRNA targets within the tissue. This was followed by multiple rounds of fluorescent probe hybridization, rolling circle amplification, high‐resolution imaging, and removal of unbound probes. Fluorescence signals corresponding to individual gene transcripts were then decoded to generate a high‐resolution spatial gene expression map at subcellular resolution. Data visualization was conducted using Xenium Explorer v3.2.0 software (10x Genomics, Pleasanton, CA, USA).

#### 4.1.6 Multiplex Immunofluorescence

mIHC staining was performed using a protocol based on Tyramide Signal Amplification (TSA) technology. Paraffin‐embedded sections were deparaffinized in xylene substitute (three changes, 10 min each), rehydrated through graded ethanol, and rinsed in distilled water. Antigen retrieval was performed in citrate buffer (pH 6.0), followed by cooling and washing with PBS (pH 7.4). Endogenous peroxidase was blocked with 3% hydrogen peroxide for 25 min, and sections were incubated with blocking buffer (10% rabbit serum for goat‐derived antibodies or 3% BSA for others) at room temperature for 30 min. Primary antibodies were then applied, followed by incubation with the corresponding HRP‐conjugated secondary antibodies and TSA reagents (Servicebio, Cat. No. G1257). Each staining step was followed by three washes in PBS for 5 min. To enable multiplexing, multiple rounds of heat‐induced epitope retrieval were performed between cycles, followed by serum blocking to prevent cross‐reactivity. Finally, sections were mounted using an antifade fluorescence mounting medium containing DAPI (Solarbio, Cat. No. C0065), and fluorescence images were acquired using a fluorescence microscope. Whole‐slide scanning was performed using the Pannoramic MIDI system (3DHISTECH, Budapest, Hungary). The primary antibodies used were as follows: Mph_SPP1 panel: CD68 (Servicebio, gb113150, 1:500), SPP1 (Proteintech, 22952‐1‐ap, 1:400); Mph_PRDM1 panel: CD68 (Servicebio, gb113150, 1:500), PRDM1 (Santa Cruz, sc‐66015, 1:100); iCAFs_HOPX panel: FAP (Abcam, ab314456, 1:200), HOPX (Santa Cruz, sc‐398703, 1:200); tCAFs_BNIP3 panel: FAP (Abcam, ab314456, 1:200), BNIP3 (Proteintech, 68091‐1‐ig, 1:400); Breast cancer epithelial panel: CK5/6 (Proteintech, 68295‐1‐ig, 1:400), MGP (Santa Cruz, sc‐271906, 1:100), SCGB2A2 (Abcam, ab150359, 1:1200).

### 4.2 Computational Methods

#### 4.2.1 Spatial Transcriptomic Sequencing Raw Data Processing

Alignment, filtering, barcode counting, and UMI counting were performed with the Space Ranger (https://www.10xgenomics.com/support/software/space‐ranger/latest) count module to generate a feature‐barcode matrix. GraphST (v1.0.0) [70] was employed to annotate epithelial cells and perform their clustering. The annotation of DCIS and IDC within the ST data was determined by integrating pathological annotations provided by pathologists.

#### 4.2.2 Single‐Cell RNAseq Raw Data Processing, Clustering and Annotation

Raw gene expression was screened using the Cell Ranger pipeline (https://www.10xgenomics.com/support/software/cell‐ranger/latest) with the reference genome GRCh38, whose results were imported by Seurat (v4.3.0) [71]. Subsequently, we used the “NormalizeData” function to normalize the expression matrix and the “FindVariableFeatures” function to determine the set of intercellular variant genes in the dataset as highly variable genes (HVGs). To eliminate the batch effect, different samples were integrated through the default parameters of the “FindIntegrationAnchors” and “IntegrateData” functions. Cells were filtered according to the following criteria: cells with < 30 000 unique molecular identifier (UMI) counts; > 200 genes and < 5500 genes; and < 10% mitochondrial gene expression in UMI counts. To perform dimensionality reduction and clustering on the expression matrix, we used “ScaleData”, and “RunPCA” was used to analyze the first 50 main components of the expression matrix. “FindNeighbors” and “FindClusters” were used to cluster the first 27 main components and project them to two‐dimensional UMAP images for display. The cell type of each group and their representative genes were decided through calculation by the “RunPrestoAll” function and previous studies.

#### 4.2.3 Copy Number Inferring for ST and scRNAseq

The CNV profiles of ST and scRNAseq data were generated using the inferCNV R package (v1.16.0) [https://github.com/broadinstitute/inferCNV]. For clone grouping, the “run” function of inferCNV was executed with the parameters: “cutoff = 0.1, cluster_by_groups = FALSE, HMM = FALSE, and denoise = TRUE”. To infer the integer copy number of each clone, the “run” function was applied again with the parameters: “cutoff = 0.1, cluster_by_groups = TRUE, analysis_mode = “cells”, and “HMM = TRUE”. For identification of malignant epithelial cells, the CNV score of each cell was defined as below:

$$\mathrm{CNV}\;\mathrm{Score}=\sum {\left(\mathrm{CN}V_{\mathrm{gene}}-1\right)}^{2}$$

The CNV score of each clone was defined as the mean CNV score of all cells belonging to that clone. Clones with a CNV score exceeding the mean CNV score of the reference group by more than one standard deviation were classified as malignant epithelial clones, and all cells within these clones were subsequently defined as malignant epithelial cells.

#### 4.2.4 Phylogenetic Reconstruction of Clonal Lineages From ST and Clone Selection

Cell‐level copy‐number states inferred by inferCNV were grouped according to clone assignments, and the median copy‐number state for each gene was calculated across cells within each clone. The processed data were then used as input for MEDICC2 to infer clonal evolution, and the resulting evolutionary relationships were visualized using ggtree (v3.8.2) [72]. The phylogenetic branches containing the closest admixture of DCIS and IDC spots were selected and defined as the matched subclones.

#### 4.2.5 scCUTseq Data Pre‐Processing and Copy Number Calling

The scCUTseq analysis pipeline was applied following the Snakemake workflows described in our previous study [21]. Fastq files were demultiplexed using a custom Python script, matching barcodes to a predefined list with up to two mismatches. Reads were assigned to single‐cell‐specific fastq files with barcodes and UMIs appended. Adapter sequences were trimmed using fastp (v0.22.0) [73], and trimmed reads were aligned to the hg19 genome with bwa‐mem (v0.7.17‐r1188) (https://github.com/lh3/bwa), then sorted and indexed using samtools (v1.10) [74]. Barcodes and UMIs were transferred from BAM headers to appropriate tags via a custom script, and duplicate reads were removed using umi‐tools (v1.1.1) [75]. Finally, copy number ratios were calculated and normalized using genomic bins of 500 kb. Genomic bins included in the blacklist (https://github.com/Boyle‐Lab/Blacklist) were removed from the analysis.

#### 4.2.6 Filtering of Low‐Quality or Diploid Cells

Low‐quality cells were removed using k‐nearest neighbor filtering as implemented in CopyKit (v0.1.2) [76]. In brief, a correlation matrix was constructed based on the copy number ratios of individual cells. For each cell, the average correlation with its five nearest neighbors was computed, and cells with an average correlation below 0.9 were excluded from further analysis. Cells with diploid profiles were identified and filtered based on the coefficient of variation (CV) of copy number ratios [6]. Post‐filtering, QC overdispersion and breadth of coverage were assessed as described [6].

#### 4.2.7 Clustering and Visualization of scCUTseq Data

Copy number ratios of individual cells were first log_2_‐transformed and then embedded in lower dimensions with UMAP using uwot (v0.1.16) [77]. Subclones were inferred through density‐based clustering with dbscan (1.1–11) [78]. Cells annotated as noise by “hdbscan” or those belonging to subclones with fewer than six cells were discarded. Heatmaps of inferCNV results and scCUTseq results were plotted with ComplexHeatmap.

#### 4.2.8 Integration Analysis of ST inferCNV and scCUTseq

To integrate inferCNV data from ST with scCUTseq data, gene‐level copy‐number profiles from inferCNV were intersected with the copy number ratios from scCUTseq. For genes located within the scCUTseq bins, values were averaged to represent the corresponding bin values in the inferCNV results. Next, both datasets were independently converted into Seurat objects, followed by “FindVariableFeatures”, “RunPCA”, and “RunUMAP”. The “FindTransferAnchors” function was then applied with ‘reduction = “cca”, dims = 1:20’, and “TransferData” was used to obtain matching scores between the two datasets. For each inferCNV clone, the scCUTseq clone with the highest corresponding score was selected as its matching clone. The corresponding CNV regions were visualized using circlize (v0.4.15) [79].

#### 4.2.9 Intrinsic Molecular Subtype Classification

Public bulk transcriptomic samples were classified using the PAM50 classifier implemented in the genefu R package (v2.32.0) based on the expression of the 50 signature genes [80].

For molecular subtyping of malignant epithelial cells in the scRNAseq datasets, we applied SCSubtype, a method specifically developed for single‐cell transcriptomic data [81], using the default parameters. Subtype‐specific scores were calculated and scaled based on established gene‐expression signatures, and each cell was assigned to the subtype with the highest score.

#### 4.2.10 Epithelial Cells NMF Analysis

To investigate possible intra‐tumoral expression programs of malignant epithelial cells in DCIS and IDC samples, we performed non‐negative matrix factorization (implemented in the geneNMF package (v0.4.0) [82]) on the malignant epithelial cells across 4 samples. Briefly, we first used “SCTransform” to normalize the expression counts, select HVGs, and run “multiNMF”. After getting the NMF programs, the method from Gavish et al. [23] was used to get robust NMF programs and meta programs: Each NMF program was defined by the top 100 genes based on NMF coefficients, and robust programs were identified using three criteria: tumor‐specific robustness, cross‐tumor robustness, and non‐redundancy. Tumor‐specific robustness required a program to appear across different K values within the same sample with ≥ 80% gene overlap. Cross‐tumor robustness was defined by ≥ 35% gene overlap with any program in other tumors. To ensure non‐redundancy, programs were ranked by similarity, selecting the most unique first and excluding others within the same tumor if they shared ≥ 60% gene overlap. Robust NMF programs were then correlated to form a correlation matrix, and NbClust (v3.0.1) [83] was used with the method of “Ward.D2” to get the best clustering number “6” by average silhouette width and total within sum of square. Then the genes of each meta‐program were ranked by their frequency among the robust NMF programs, and the top 100 were selected as the marker genes of meta‐programs. The marker genes were used in the function “AddModuleScore_UCell” from UCell (v2.4.0) [84] and “runUMAP” described from geneNMF. Jaccard similarity was then calculated to decide the classification of the malignant epithelial cell subpopulations. The thresholds to distinguish MP levels were based on the “AUCell_exploreThresholds” function from AUCell (v1.22.0) [85].

#### 4.2.11 Pathway Enrichment Analysis and Calculation of Gene Signature Scores

For NMF programs, “runGSEA” from geneNMF was utilized to analyze enriched pathways across individual programs. For MP subpopulations of malignant epithelial cells, the GSVA (v1.48.3) [86] package was employed to investigate subpopulation‐specific pathway enrichment, with results visualized through heatmaps generated by ComplexHeatmap (v2.16.0) [87]. For DCIS and IDC, as well as immune cells and stroma cells, “RunPrestoAll” was first used to identify DEGs, which were then utilized as input for “GSEA”, “enrichGO” and “enrichKEGG” functions using ClusterProfiler (v4.8.2) [88]. Function “AddModuleScore_UCell” was used for calculating gene sets signature scores of malignant epithelial cells, immune cells and stroma cells.

#### 4.2.12 Pseudotime Trajectory Analysis and RNA Velocity Analysis

Monocle3 (v1.3.1) [89] was employed to infer developmental trajectory relationships among malignant epithelial cells and CAF subpopulations. The trajectory graph was inferred using the “learn_graph” function with ncenter = 500. Meanwhile, slingshot (v2.8.0) [90] was implemented for trajectory inference with approx_points = 150. For RNA velocity analysis, velovi (v0.3.1) [91] was utilized with spliced and unspliced matrices that underwent initial normalization and filtering to retain robust genes. Moment calculations of normalized spliced/unspliced counts were performed via the “scv.pp.moments” function. Full transcriptional dynamics were subsequently reconstructed using the “VELOVI” function, followed by RNA velocity estimation via the “scv.tl.velocity_graph” function. Finally, velocity patterns were visualized on malignant epithelial cell subpopulation UMAP embeddings using scv.pl.velocity_embedding_stream.

#### 4.2.13 SCENIC Analysis

TF activity of malignant epithelial cells was analyzed using pySCENIC (v0.12.1) [92], with raw count matrices as input. The pySCENIC pipeline was used to infer regulons and compute TF activity scores for each cell. Differentially activated TFs in each subpopulation were identified by calculating subpopulation‐specific fold changes. The top five differentially activated TFs were visualized in a dot plot.

#### 4.2.14 Survival Analysis of HTAN Database

The CNV profiles, bulk RNAseq expression data, and corresponding clinical information for pure DCIS samples from the TBCRC and RAHBT cohorts were obtained from the HTAN database [18, 93]. Basal‐like samples were not included based on PAM50 subtypes, resulting in a total of 197 samples for subsequent analyses. To integrate the scRNAseq data with the HTAN cohort, BayesPrism (v2.2.2) [94] was employed to estimate the activities of MPs and the proportions of other cell subpopulations in each bulk RNAseq sample. Patients were subsequently stratified into high‐ and low‐score groups using the optimal cutoff determined by the “surv_cutpoint” function in the survminer package (v0.4.9) (https://github.com/kassambara/survminer). Kaplan–Meier survival curves were visualized using the “ggsurvplot” function from survminer.

#### 4.2.15 Re‐Clustering of Immune Cells and Stroma Cells

For immune cell subpopulations, cells were first extracted from the integrated overview dataset. After extraction, the dataset was processed through scaling, PCA, and clustering, as described above. B cell clustering began with the initial classification into B cells and plasma cells (B_IgG) based on *MS4A1* and *IGHG1* expressions. B cells were further divided into memory and naïve subpopulations using *IGHD*, while plasma cells were categorized into mature and immature based on *PRDM1* expression. T cells were initially separated into CD4^+^ and CD8^+^ populations and subsequently annotated using published gene signatures. Among the myeloid cells, we identified monocytes (*FCN1*, *VCAN*), dendritic cells (*ITGAX*), neutrophils (*FCGR3B*, *S100A8*, *G0S2*), and macrophages (*CD68*, *MSR1*, *CD163*). Subpopulations within macrophages and dendritic cells were annotated based on their marker genes and previously reported classifications. Endothelial and perivascular subpopulations were further annotated based on marker genes reported in a previous study [95]. CAF subpopulations were annotated and named based on their functional enrichment patterns and representative marker genes, with reference to previously reported classifications [65, 96, 97]. Cell differentiation scores were determined using CytoTRACE2 (v1.0.0) [34], with raw counts and default parameters.

#### 4.2.16 Integration Analysis of ST and scRNAseq

Cell2location (v0.1.4) [15], CelEry (v1.1.2) [27] and CellTrek (v0.0.94) [14] were applied as described previously. Cell2location was used to spatially map the cell types annotated in the scRNAseq data onto the ST data by implementing a negative binomial regression model. The reference signature of annotated cell types was estimated at two hierarchical levels: (1) broad cell categories, including epithelial cells, T/NK cells, CAFs, endothelial cells, perivascular cells, myeloid cells, B cells, and mast cells; and (2) fine‐grained subpopulations within each major category. An unnormalized mRNA count matrix was used as input, with sample IDs treated as batch variables. The model was trained with max_epochs = 500 and train_size = 1. The trained reference signature model was then used by cell2location to estimate the spatial abundance of cell types, using 30,000 training iterations and a train_size of 1. The distribution of broad cell categories in the ST data was visualized, while fine‐grained subpopulation mapping results were utilized for further analyses. For CelEry, differential analysis was performed on ST regions annotated as DCIS and IDC, identifying DEGs with *p* < 0.001 and log_2_Foldchange > 0.25, which were subsequently used for integrative analysis. Following the established CelEry pipeline, malignant epithelial cell scRNAseq data and ST data containing only DEGs were processed and filtered using “cel.get_zscore” and “cel.drop_NaN”, retaining only the shared genes between the two datasets. Domain weights were computed according to the recommended methodology, followed by domain fitting using “cel.Fit_domain” with “num_epochs_max = 500” and “batch_size = 778”. Finally, cell type prediction was performed using “cel.Predict_domain” with “class_num = 2” to classify cells into DCIS‐ or IDC‐associated subpopulations. To analyze ST data using CellTrek, we first applied the function “traint” to co‐embed the scRNAseq and ST data into a shared feature space, using default parameters and considering fine‐grained subpopulations within each major category. We then ran “celltrek” on the co‐embedded data with the following parameters: “intp_pnt = 5000, nPCs = 30, ntree = 1000, top_spot = 5, spot_n = 20, and repel_r = 20”, with a total of 20 iterations.

#### 4.2.17 Calculation of Spatial Distance and Spatial Distribution

We calculated spatial distance and spatial distribution in both CellTrek and cell2location. Spatial distances were assessed in two aspects: (1) the pairwise spatial distance between cells mapped to ST spots, and (2) the distance between individual ST spots and the annotated boundaries of DCIS or IDC regions. Both were computed using the “RadialDistance” function from the semla package (v1.1.6) [98]. Cells with a negative distance from the borderline were considered within the region, those with a distance between 0 and 200 µm were defined as the border area, and those with a distance greater than 200 µm were classified as stroma area. For CellTrek, ORs were calculated for each subpopulation in DCIS or IDC areas to quantify its tissue preference, as previously reported [99]. A contingency table was constructed using the number of subpopulations in DCIS, IDC, their border areas, and the stroma area. Fisher's exact test was then applied to calculate ORs and corresponding *P* values, which were adjusted using the Benjamini‐Hochberg (BH) method. Significant enrichment or depletion was defined as an adjusted *p* < 0.01 and OR > 1.2 or < 0.8. Cell subpopulations that were enriched in DCIS but depleted in IDC, and vice versa, were selected for further analysis. mCAFs_THBS1 was also considered, which was exclusively enriched in the DCIS border, while Mph_MKI67 and CD4_Proliferation were excluded because of their high expression of cell cycle signature. The selected cell subpopulations were further analyzed using cell2location to examine their spatial distribution. The average cell2location frequencies for these cell subpopulations or gene set scores at the same distances were aligned and stacked by DCIS or IDC, extending from the tumor center to the external stroma. The spatial distances between myoepithelial cells and CAFs were calculated using the phenoptr R package (0.3.2) (https://akoyabio.github.io/phenoptr/).

#### 4.2.18 BCR Repertoire Profiling

The spatial distribution of BCR clonotypes and the distances between mRNA VH CDR3 sequences were analyzed as previously described [100]. In the circular tree representation, IGH clonotypes are depicted as the leaves of the tree, labeled by their translated VH CDR3 sequence and the clonal family they belong to (with “0” indicating no clonal membership).

#### 4.2.19 Receptor‐Ligand Interaction Prediction

To predict signaling crosstalk between selected cell subpopulations and DCIS or IDC, we utilized CellChat (v2.1.1) [29], which can infer cell‐cell communication from multiple spatially resolved transcriptomics datasets. ST datasets from 4 samples were merged using the “createCellChat” function, following the recommended workflow. Cellular communication probabilities were then computed with “computeCommunProb”, setting “interaction.range = 250” and “contact.range = 100”. Statistically and biologically significant interactions were identified, with the top 10 most prominent interactions visualized using “netVisual_bubble”.

#### 4.2.20 Integration Analysis of scRNAseq and Xenium

Raw data from the Xenium instrument, including feature‐cell matrices and cell boundary files, were integrated into Seurat using default parameters. Cell type annotation in Xenium data was inferred using the RCTD algorithm from the spacexr R package (v2.2.1) [16], leveraging scRNAseq data as a reference. The analysis followed the recommended Seurat spatial integration pipeline (https://satijalab.org/seurat/). DCIS and IDC regions were annotated based on pathological assessments provided by pathologists. The spatial distributions of specific macrophage and CAF subpopulations were visualized using spatialdata_plot (v0.2.9) [101].

#### 4.2.21 Machine‐Learning Model Construction and Validation

We developed an integrated machine‐learning framework to construct a robust classifier for distinguishing DCIS from IDC. The 100 genes defining each of MP1 and MP5 (Table S2), together with the top 100 differentially expressed genes from Mph_SPP1, Mph_PRDM1, iCAFs_HOPX, and tCAFs_BNIP3 ranked by average log2 fold change (Table S4), were included as candidate molecular features. These candidate features were independently selected using five feature‐selection algorithms: random forest (RF), least absolute shrinkage and selection operator (Lasso), gradient boosting of generalized linear models (glmBoost), and stepwise generalized linear models with bidirectional or backward selection (Stepglm[both] and Stepglm[backward], respectively). The complete feature set without prior feature selection was additionally retained, resulting in six candidate feature sets.

Each feature set was subsequently evaluated using 21 classification algorithms: RF, tree‐based extreme gradient boosting (XGBTree), radial‐kernel support vector machine (SVM), logistic regression (LR), k‐nearest neighbors (KNN), partial least squares (PLS), gradient boosting machine (GBM), neural network (NN), naive Bayes (NB), linear discriminant analysis (LDA), Lasso, ridge regression, elastic net, adaptive boosting (AdaBoost), linear XGBoost (XGBLinear), stepwise generalized linear models with bidirectional or forward selection (Stepglm[both] and Stepglm[forward], respectively), glmBoost, partial least squares generalized linear regression (plsRglm), CatBoost, and LightGBM. After excluding combinations in which the same or closely related algorithm was used for both feature selection and model construction, 119 nonredundant model configurations were generated.

Model training and hyperparameter optimization were performed using five‐fold cross‐validation. Algorithm‐specific hyperparameters were optimized through grid search. Model performance was assessed using the area under the receiver operating characteristic curve (AUC) derived from cross‐validated predictions in the training dataset and predictions in four independent validation cohorts. For each model configuration, the mean AUC and its standard deviation were calculated across the training dataset and the four independent validation cohorts. The best‐performing model for each feature set was identified according to the highest mean AUC, and all model configurations were subsequently ranked to determine the overall optimal model.

For interpretation of the glmBoost+PLS model, feature importance was quantified using the model‐specific variable‐importance method implemented in the caret package (6.0–94) [102]. Candidate genes were ranked according to their relative importance scores, and the 10 highest‐ranked genes were selected for downstream interpretation.

To further interpret the optimal glmBoost+PLS model, SHapley Additive exPlanations (SHAP) values were estimated using the model‐agnostic Kernel SHAP algorithm implemented in the kernelshap package (0.9.1) [103]. SHAP values were calculated for the training samples using the model‐predicted probability of IDC as the output. Global feature importance was quantified as the mean absolute SHAP value of each gene across all samples. The distribution and direction of gene contributions were visualized using SHAP beeswarm plots generated with the shapviz package (0.10.3) [104]. Positive SHAP values indicated contributions toward an IDC prediction, whereas negative values indicated contributions toward a DCIS prediction. Analyses focused on the 10 highest‐ranked genes identified by the model‐specific variable‐importance analysis.

#### 4.2.22 Virtual Gene Knockout Analysis at Single‐Cell Level

To investigate the regulatory effects of *MGP*, *ERBB2*, *SERPINA3*, *RBP1*, and *TPX2*, we performed virtual gene knockout analyses using scTenifoldKnk and Geneformer on scRNAseq data from pure DCIS samples [20, 105, 106]. scTenifoldKnk was run with the following parameters: nc_nNet = 10, nc_nCells = 500, qc_mtThreshold = 0.1, and qc_minLSize = 1000. Geneformer was applied to the same DCIS scRNAseq dataset using the pretrained Geneformer‐V2‐104 m model. Genes with a fold change greater than 1 in the scTenifoldKnk analysis and an embedding shift above the 75th percentile in the Geneformer analysis were considered significantly perturbed. Genes meeting these criteria in both analyses were subsequently subjected to functional enrichment analysis.

#### 4.2.23 Quantification and Statistical Analysis

All statistical analyses were performed using R (v4.4.1) and Python (with version selection based on software requirements). Unless otherwise noted, two‐group comparisons were carried out with the two‐sided Wilcoxon rank‐sum test applied to unpaired data. Detailed descriptions of all statistical methods used in this study are provided in the corresponding sections of the methods and specific figure legends.

## Author Contributions

Project conceptualization and design: Q.Y., N.Z. scCUTseq application: N.Z., S.Z. Breast cancer sample collection: B.C., W.Z., L.W., J.H. and H.W. Breast cancer sample annotation: P.S., N.Z. Data analysis and visualization: T.W., Y.J. Multi‐omics analysis: J.L., Z.C. CelEry algorithm application: Q.Z. Xenium application: Z.D. Funding acquisition: Q.Y., N.Z. Project coordination: N.Z. Figures: N.Z., T.W., S.Z. Writing: N.Z., T.W., S.Z. with input from all the authors. Manuscript revision: T.X., U.S., N.C., B.L. and Q.Y.

## Conflicts of Interest

The authors declare no conflicts of interest.

## Supporting information




**Supporting File 1**: advs77897‐sup‐0001‐SuppMat.pdf.

**Supporting File 2**: advs77897‐sup‐0002‐TableS1.xlsx.

**Supporting File 3**: advs77897‐sup‐0003‐TableS2.xlsx.

**Supporting File 4**: advs77897‐sup‐0004‐TableS3.xlsx.

**Supporting File 5**: advs77897‐sup‐0005‐TableS4.xlsx.

## Data Availability

Publicly available bulk transcriptomic datasets were retrieved primarily from the Gene Expression Omnibus (GEO) and supplemented with data from the INVADE study. The training cohort comprised GSE41228, GSE21422, GSE69240, GSE87517, GSE7882, GSE66301, GSE3893, and GSE33692, whereas the independent validation cohorts included GSE26304, GSE59246, GSE72205, and the INVADE cohort. CNV profiles, bulk RNAseq data, and corresponding clinical information for pure DCIS samples from the TBCRC and RAHBT cohorts, together with the pure DCIS scRNAseq dataset, were accessed through the HTAN (https://humantumoratlas.org/). The raw sequence data of scRNAseq, ST, scCUTseq, and Xenium generated in this study have been deposited in the Genome Sequence Archive for Human (GSA‐Human; https://ngdc.cncb.ac.cn/bioproject/browse/PRJCA041870) under accession number PRJCA041870.
