# Supplementary material for: Integrated Multi‐Omics Reveals Cellular States and Microenvironmental Remodeling in Coexisting DCIS and IDC
Source: Adv Sci (Weinh). 2026 Sep 27:e77897. Online ahead of print. doi: 10.1002/advs.77897 (PMC13616352; doi:10.1002/advs.77897)
Supplement: Supplementary file 1 [file ADVS-9999-e77897-s005.pdf]

SUPPLEMENTARY FIGURES

Integrated Multi-Omics Reveals Cellular States and Microenvironmental Remodeling in Coexisting DCIS and IDC

Ning Zhang[1], Tong Wan[1], Siyue Zhang[1,2], Yunzhen Jiang[3], Peng Su[4], Jixin Liu[5], Zhitong Chen[5], Jie Hao[1], Haoyu Wang[1], Bing Chen[6], Wenjing Zhao[6], Lijuan Wang[6], Tao Xing[7,8], Qihuang Zhang[9], Ulf Schmitz[10,11,12], Zhiyong Ding[13], Nicola Crosetto[14,15,16], Bingqiang Liu[5], Qifeng Yang[1,6,17]

[1]Department of Breast Surgery, General Surgery, Qilu Hospital of Shandong University, Ji'nan, China

[2]Department of General Surgery, Beijing Tiantan Hospital, Capital Medical University, Beijing, China

[3]Cheong Kun Lun College, University of Macau, Macau, China

[4]Department of Pathology, Qilu Hospital of Shandong University, Ji'nan, China

[5]School of Mathematics, Shandong University, Ji'nan, China

[6]Biological Resource Center, Qilu Hospital of Shandong University, Ji'nan, China

[7]Department of Radiation Oncology, Townsville University Hospital, Townsville, Australia

[8]College of Medicine and Dentistry, James Cook University, Townsville, Australia

[9]Department of Epidemiology, Biostatistics and Occupational Health, School of Population and Global Health, McGill University, Montreal, Canada

[10]Computational Biomedicine Lab, College of Science and Engineering, James Cook University, Townsville, Australia

[11]Centre for Tropical Bioinformatics and Molecular Biology, James Cook University, Cairns, Australia

[12]Centenary Institute, The University of Sydney, Camperdown, Australia

[13]Mills Institute for Personalized Cancer Care, Fynn Biotechnologies Ltd., Ji'nan, China

[14]Department of Microbiology, Tumor and Cell Biology, Karolinska Institutet, Stockholm, Sweden

[15]Science for Life Laboratory, Stockholm, Sweden

[16]Human Technopole, Milan, Italy

[17]Research Institute of Breast Cancer, Shandong University, Ji'nan, China

These authors contributed equally: N.Z, T.W, S.Z.

Correspondence to Q.Y. (qifengy_sdu@163.com)

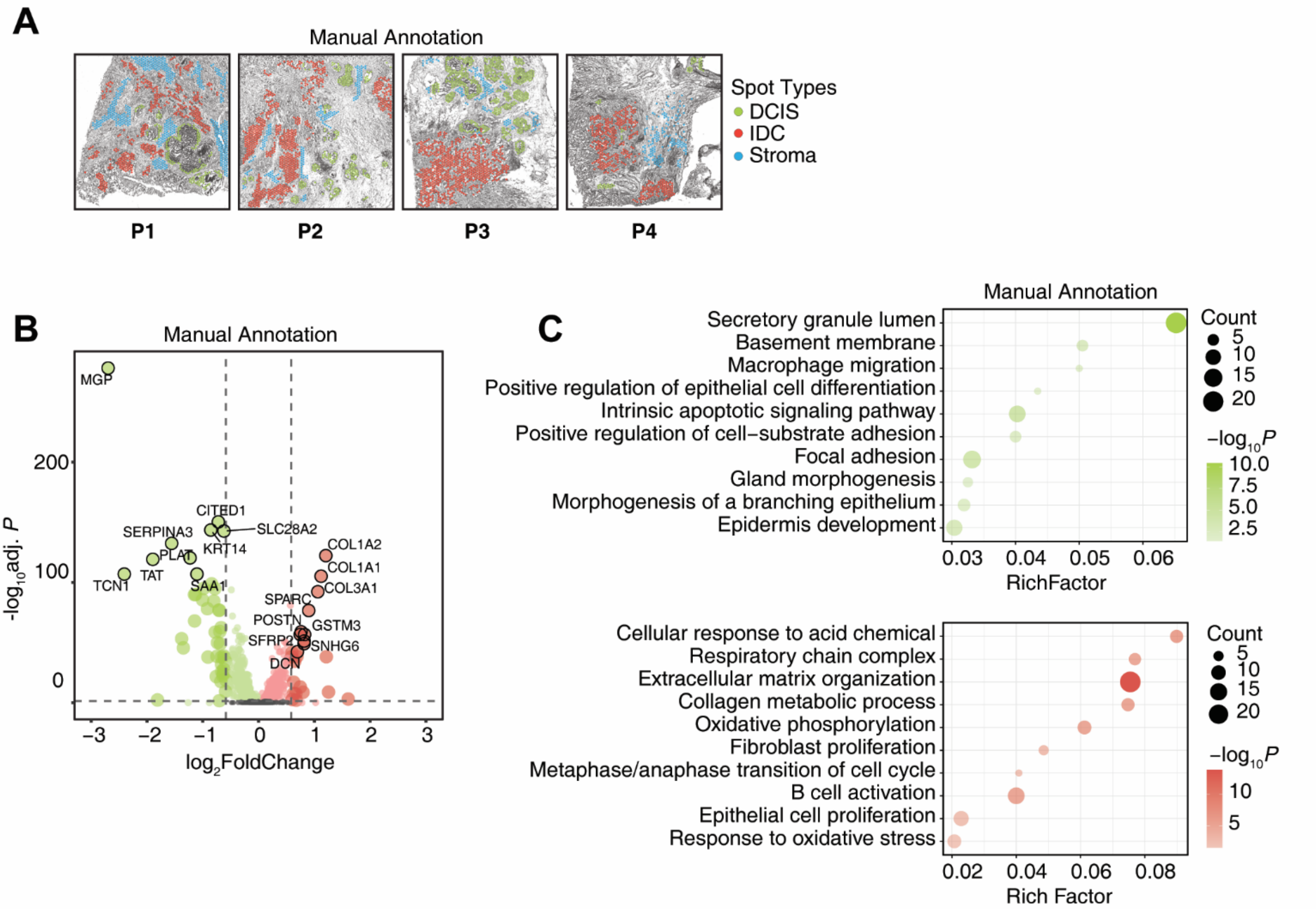

Supplementary Figure 1. Differentially expressed genes and functional pathways between DCIS and IDC from spatial transcriptomics by manual annotation. Related to Figure 1.

(A) Spatial overview of manually annotated DCIS and IDC regions by the pathologist across four samples. (B) DEGs between DCIS and IDC in ST annotated as shown in (A), with the top 10 genes highlighted by black circles. (C) GO pathway enrichment analysis for DCIS (top panel) and IDC (bottom panel) in ST with manual annotation.

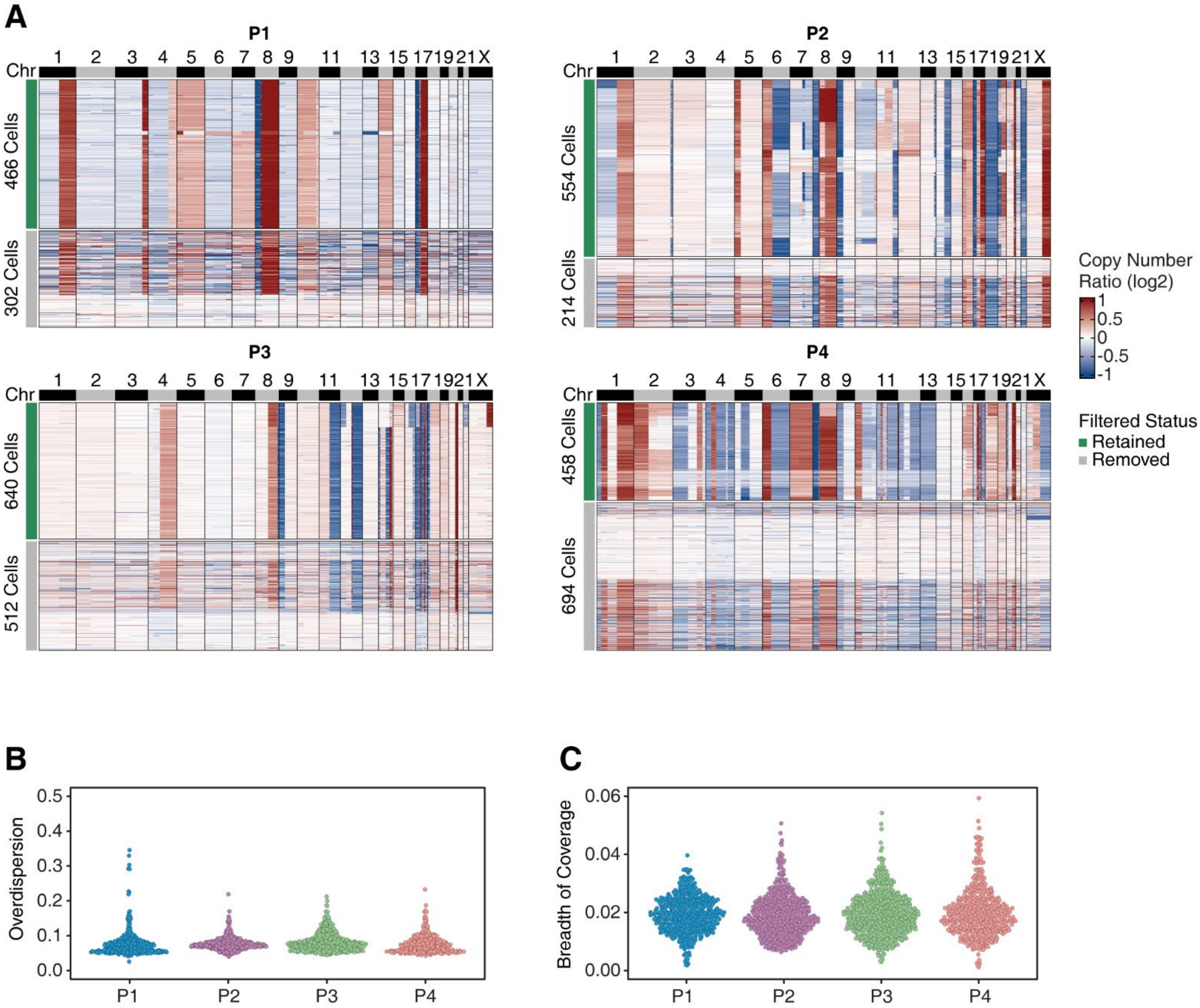

Supplementary Figure 2. Quality control of scCUTseq. Related to Figure 2.

(A) Heatmaps of CNV profiles for single cells obtained from scCUTseq across four samples. Left annotations indicate retained or removed cells. Chromosome Y was excluded (B) Comparison of overdispersion metrics for genomic bin counts across four samples. (C) Breadth of coverage metrics for four samples.

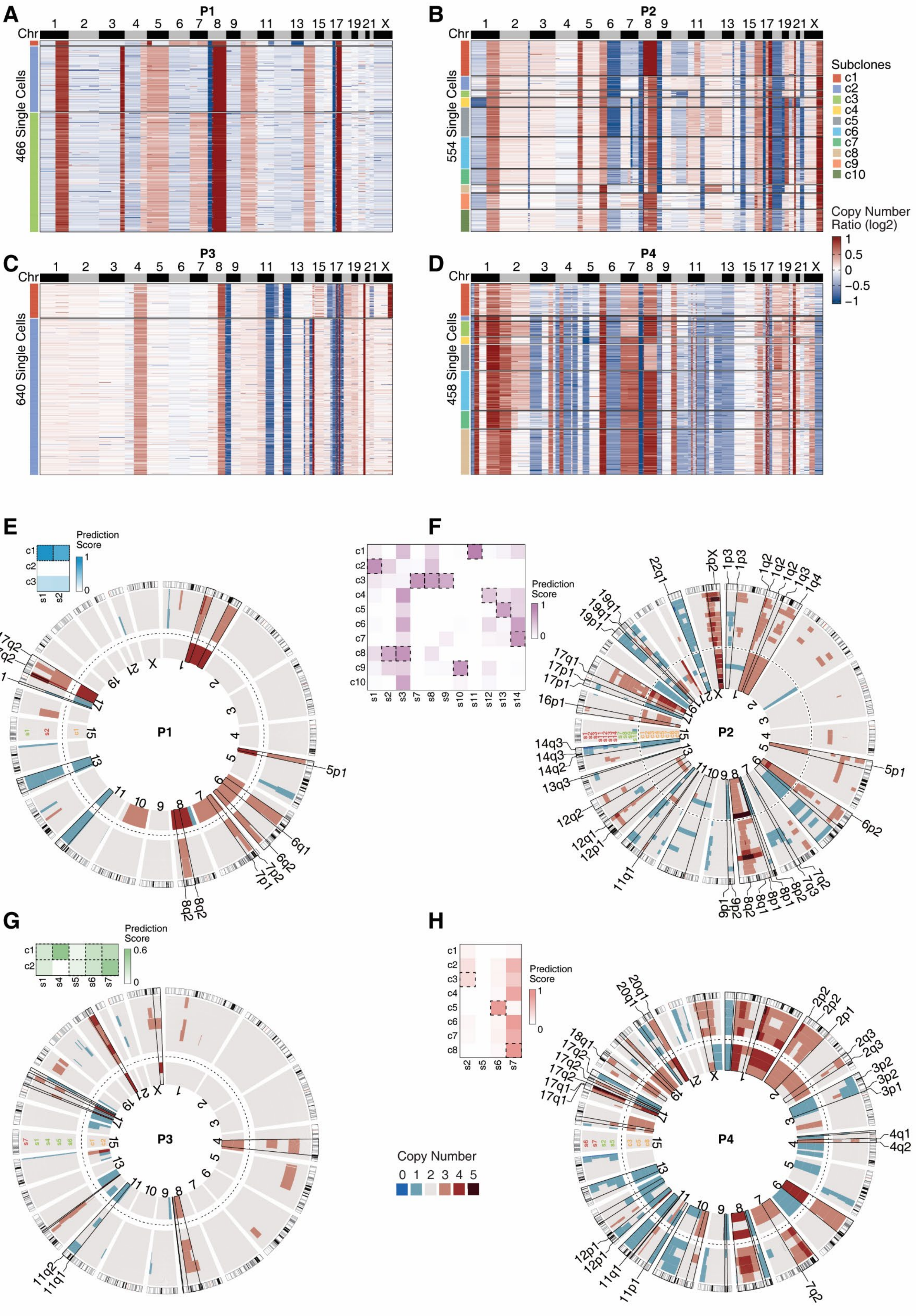

A
B
C
D
E
F
G
H
P1
P2
P3
P4
Chr
466 Single Cells
554 Single Cells
640 Single Cells
458 Single Cells
Subclones
c1
c2
c3
c4
c5
c6
c7
c8
c9
c10
Copy Number Ratio (log2)
Prediction Score
Copy Number
0 1 2 3 4 5

Supplementary Figure 3. CNV profiling by integration of scCUTseq and spatial transcriptomics. Related to Figure 2.

(A, B, C, D) Heatmaps of CNV profiles for retained individual cells from scCUTseq across four samples. The left annotation columns indicate subclonal identities. (E, F, G, H) Circular plots illustrating CNV profiles of selected subclones from ST and scCUTseq data across four samples. The heatmap in the upper-left corner of each circular plot displays the correspondence scores between subclones from ST and scCUTseq across four samples, with black dashed rectangles highlighting the highest scores. Clone names in red indicate IDC subclones from ST, those in green indicate DCIS subclones from ST, and those in orange indicate scCUTseq-derived subclones. Among the cytobands with CNV events, those labeled indicate CNVs present in IDC but absent from DCIS. Chromosomal cytobands are shown on the outer ring. Chromosome Y was excluded.

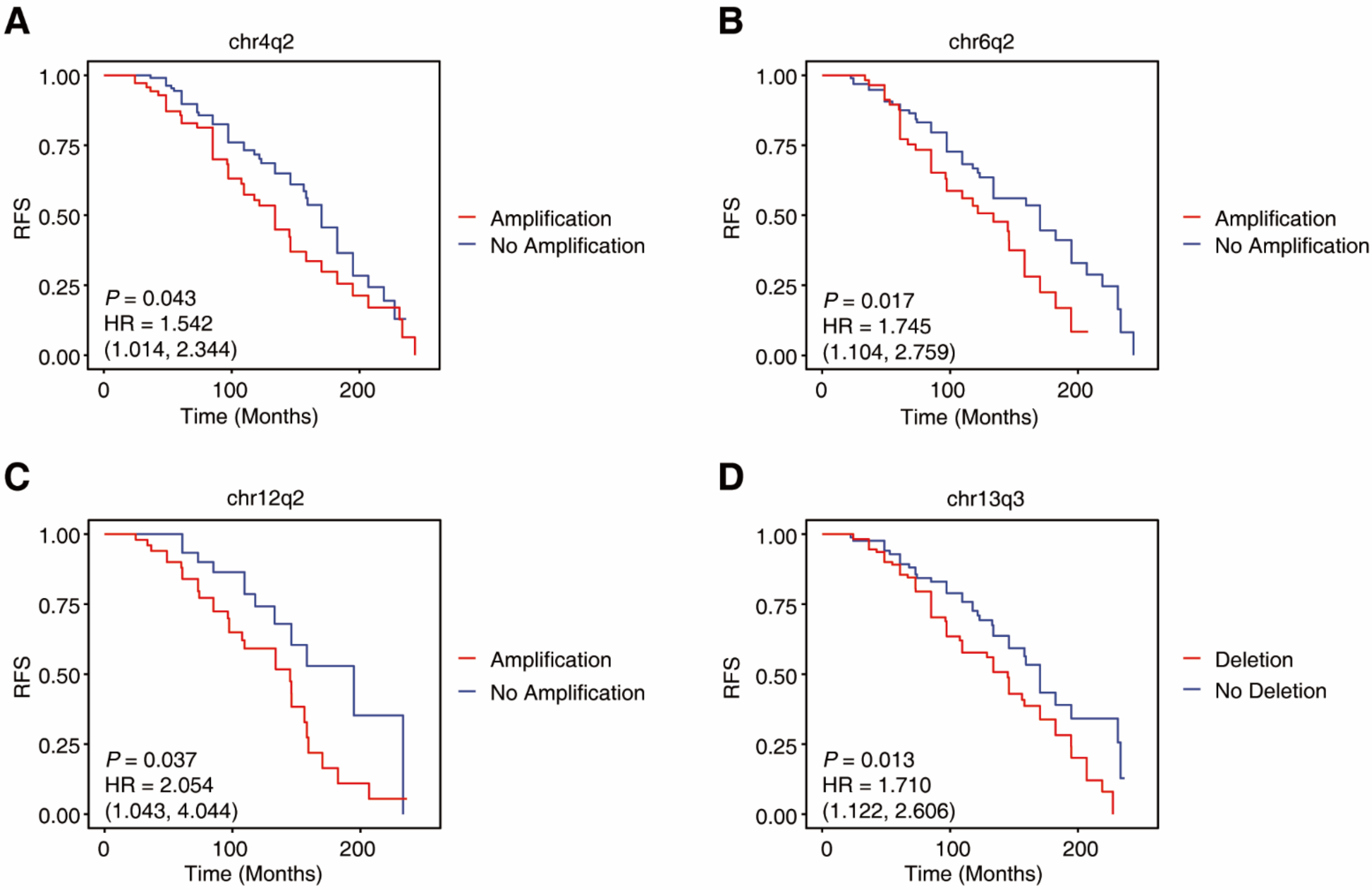

Supplementary Figure 4. RFS outcomes associated with CNV events. Related to Figure 2.

(A, B, C, D) Kaplan-Meier survival curves illustrating RFS outcomes associated with amplification of chr4q2, chr6q2, chr12q2 and deletion of chr13q3 based on CNV profiles from the HTAN bulk DNA cohort. Significance was assessed using the log-rank test. HR and 95% CI were estimated using univariable Cox proportional hazards regression.

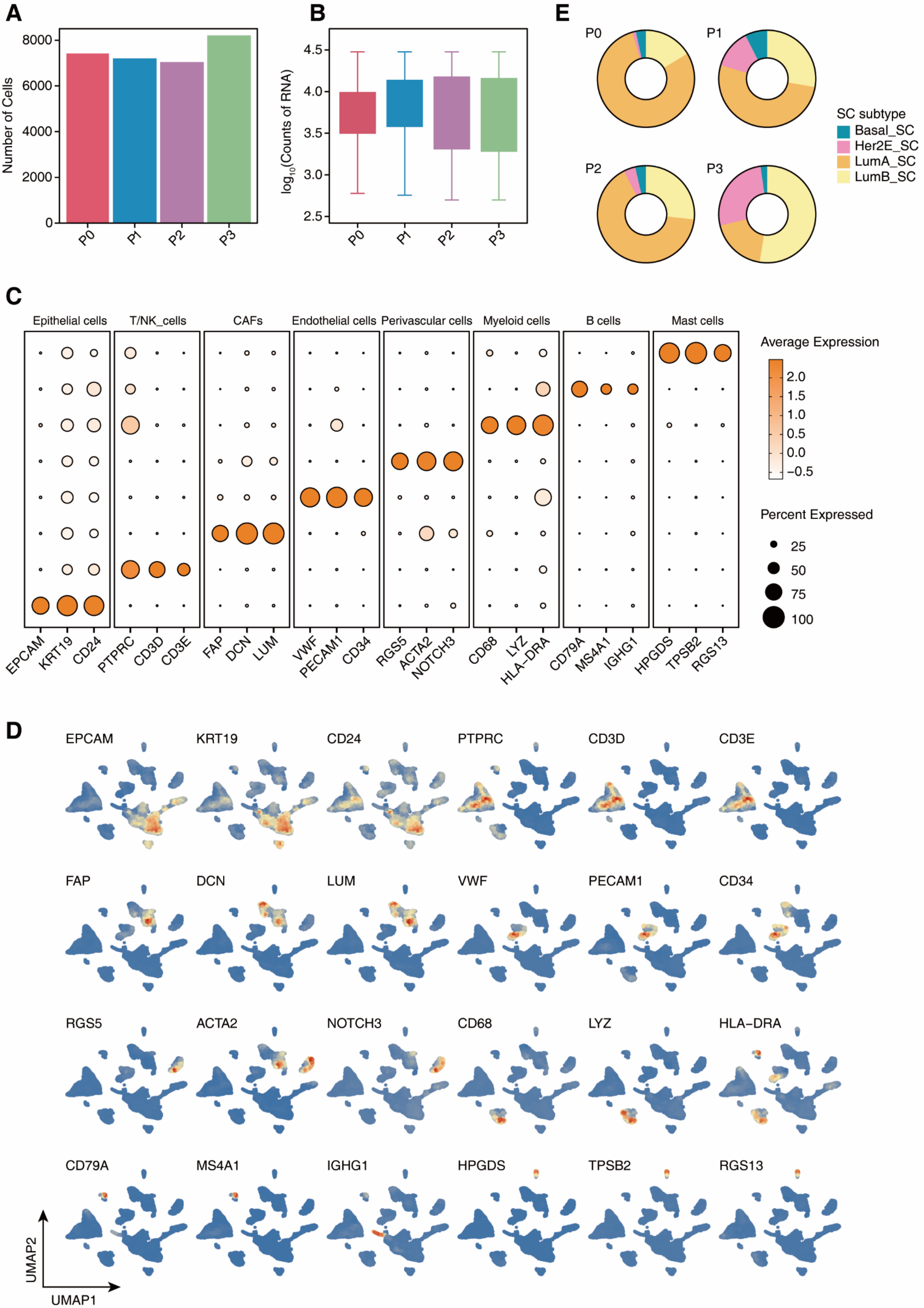

A
Number of Cells
0
2000
4000
6000
8000
P0
P1
P2
P3
B
log$_{10}$(Counts of RNA)
2.5
3.0
3.5
4.0
4.5
P0
P1
P2
P3
E
P0
P1
P2
P3
SC subtype
Basal_SC
Her2E_SC
LumA_SC
LumB_SC
C
Epithelial cells
T/NK_cells
CAFs
Endothelial cells
Perivascular cells
Myeloid cells
B cells
Mast cells
Average Expression
2.0
1.5
1.0
0.5
0.0
−0.5
Percent Expressed
25
50
75
100
EPCAM
KRT19
CD24
PTPRC
CD3D
CD3E
FAP
DCN
LUM
VWF
PECAM1
CD34
RGS5
ACTA2
NOTCH3
CD68
LYZ
HLA−DRA
CD79A
MS4A1
IGHG1
HPGDS
TPSB2
RGS13
D
EPCAM
KRT19
CD24
PTPRC
CD3D
CD3E
FAP
DCN
LUM
VWF
PECAM1
CD34
RGS5
ACTA2
NOTCH3
CD68
LYZ
HLA−DRA
CD79A
MS4A1
IGHG1
HPGDS
TPSB2
RGS13
UMAP2
UMAP1

Supplementary Figure 5. Overview of single-cell transcriptomic profiling of samples with synchronous DCIS and IDC. Related to Figure 3.

(A) Number of cells detected in each scRNAseq sample. (B) log10-transformed UMI counts for each scRNAseq sample. (C) Bubble plot visualizing the expression patterns of key marker genes across cell types. Dot size corresponds to the fraction of cells expressing each gene, whereas color intensity represents the normalized expression level. (D) UMAP plots depicting the distribution of marker genes for each cell type. (E) Distribution of patient-derived cells across molecular subtypes assigned using SCSubtype.

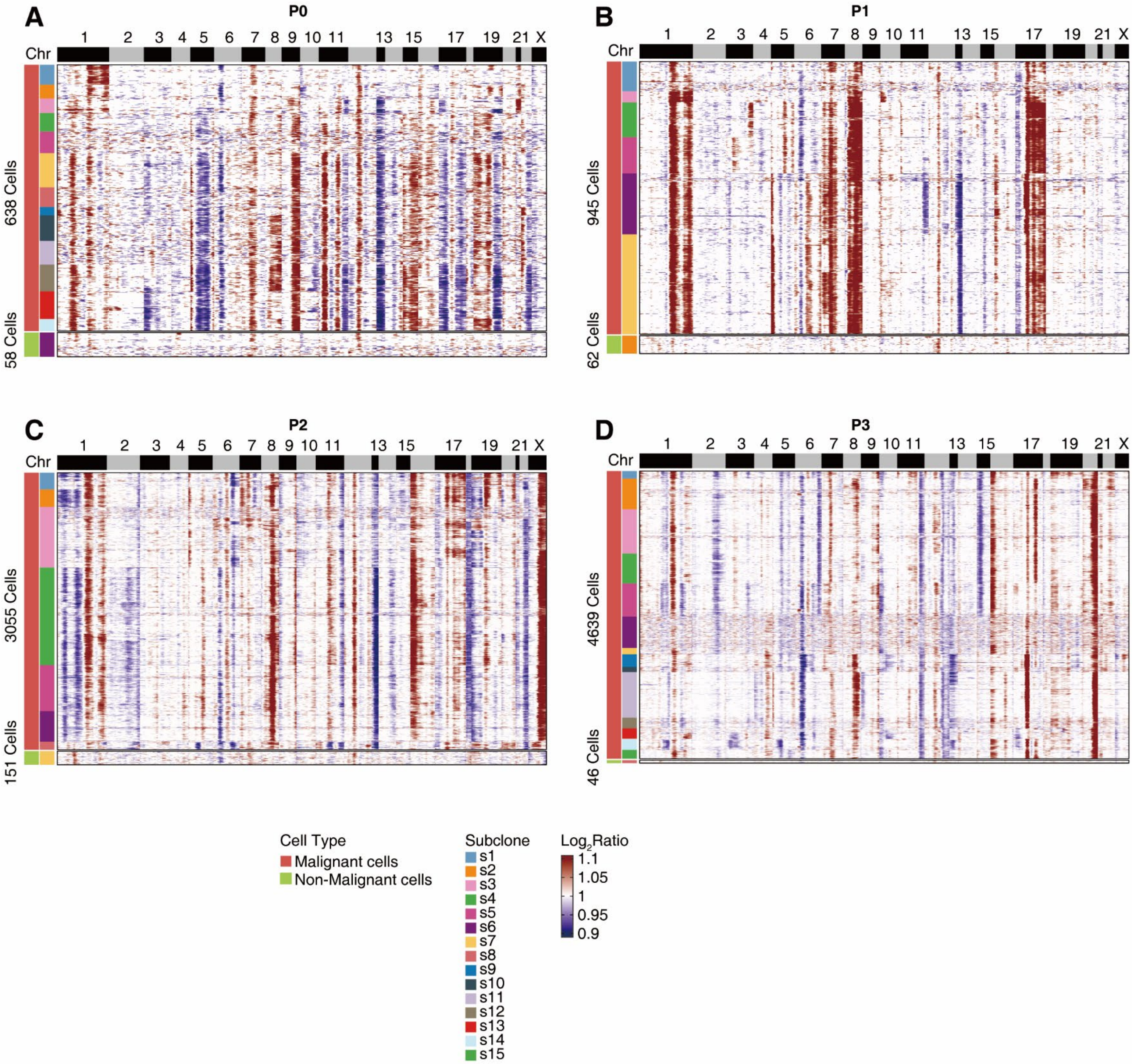

Supplementary Figure 6. CNV profiles inferred from scRNAseq. Related to Figure 3.

(A, B, C, D) Heatmaps of inferred CNV profiles for single cells (rows) from scRNAseq across four samples. Left annotations indicate malignant status and subclonal identities. Non-malignant cells were removed from subsequent analyses.

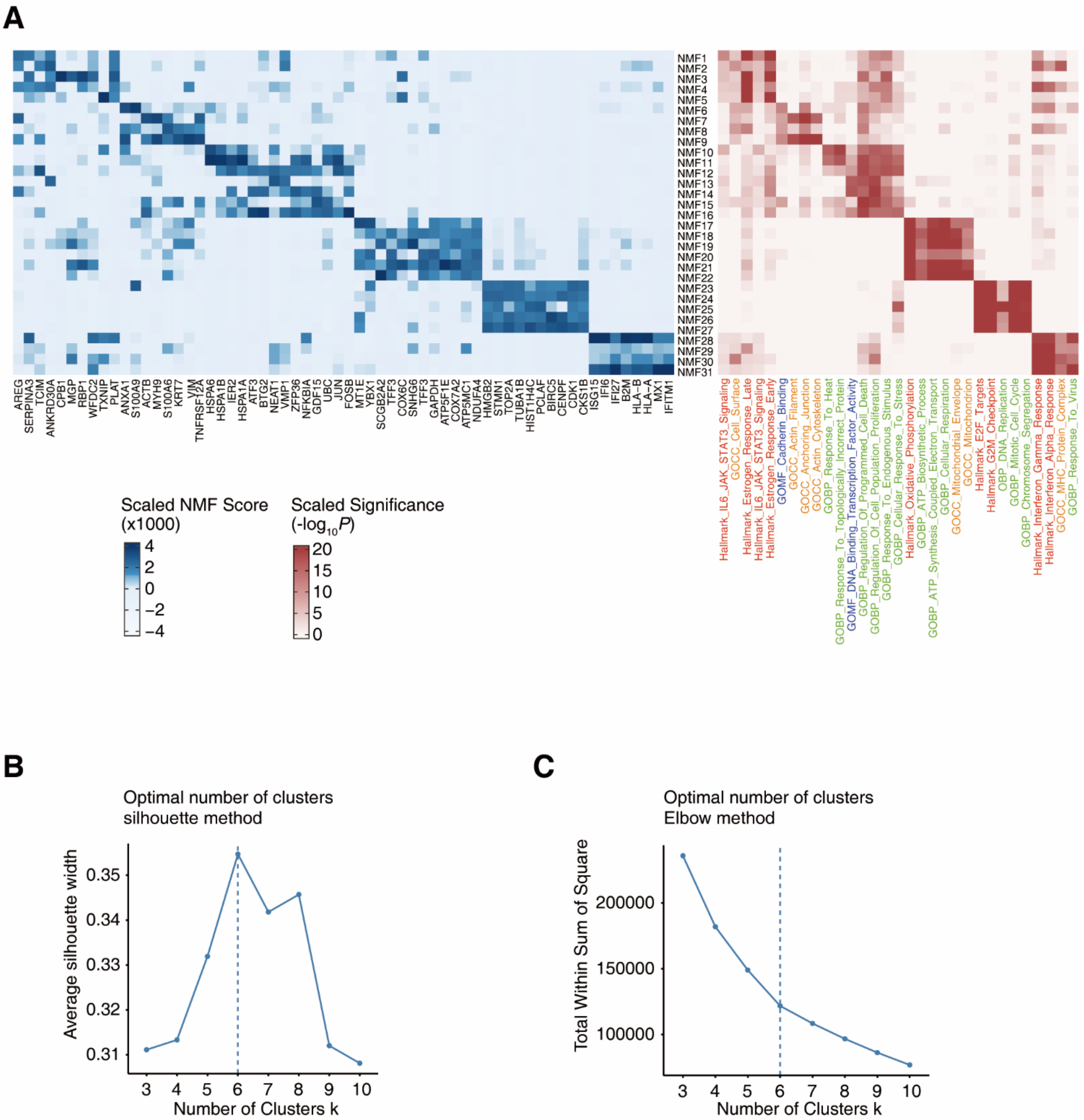

Supplementary Figure 7. Optimal cluster numbers, representative genes and pathways of MPs. Related to Figure 3.

(A) Left panel: Heatmap displaying scaled NMF scores for representative MP genes (columns) across all robust NMF programs (rows). MP numbers are indicated on the right, and selected genes are labeled at the bottom. Right panel: Heatmap showing scaled significance of enrichment, shown as scaled -log$_{10}$$P$, for selected functional annotations (columns) across all MPs (rows, ordered as in the left panel), with key functional annotations labeled at the bottom. (B) Average silhouette width for different numbers of MPs, with the optimal number indicated by a blue dashed vertical line. (C) Within-cluster sum of squares for different numbers of MPs, with the optimal number indicated by a blue dashed vertical line.

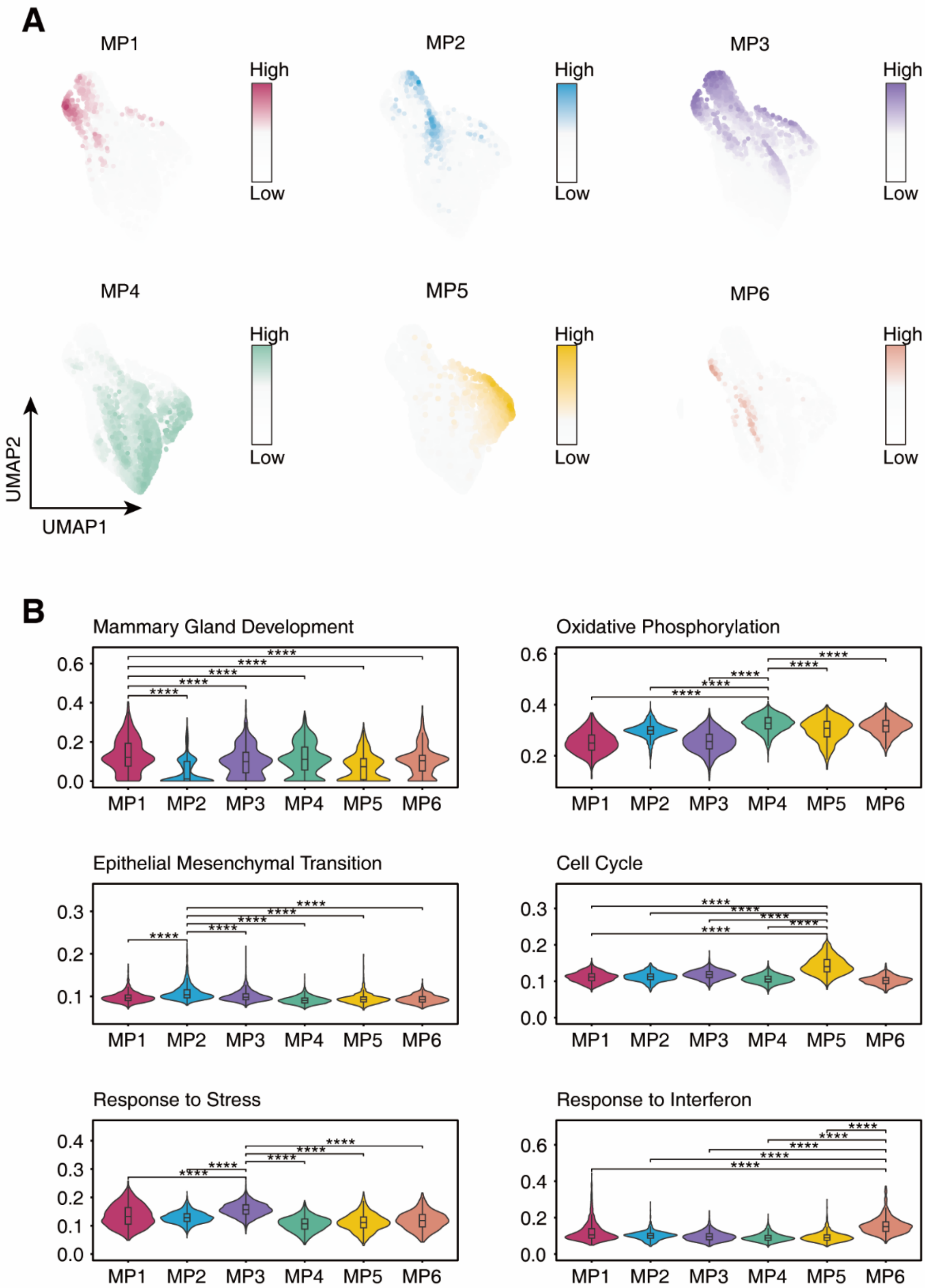

Supplementary Figure 8. Scores and functional annotations of MPs. Related to Figure 3.

(A) UMAP plots showing MP scores across malignant epithelial cells. (B) Violin plots depicting representative pathway scores among malignant epithelial cells classified by MPs. Statistical significance was assessed using a two-sided Wilcoxon test; *****P* < 0.0001.

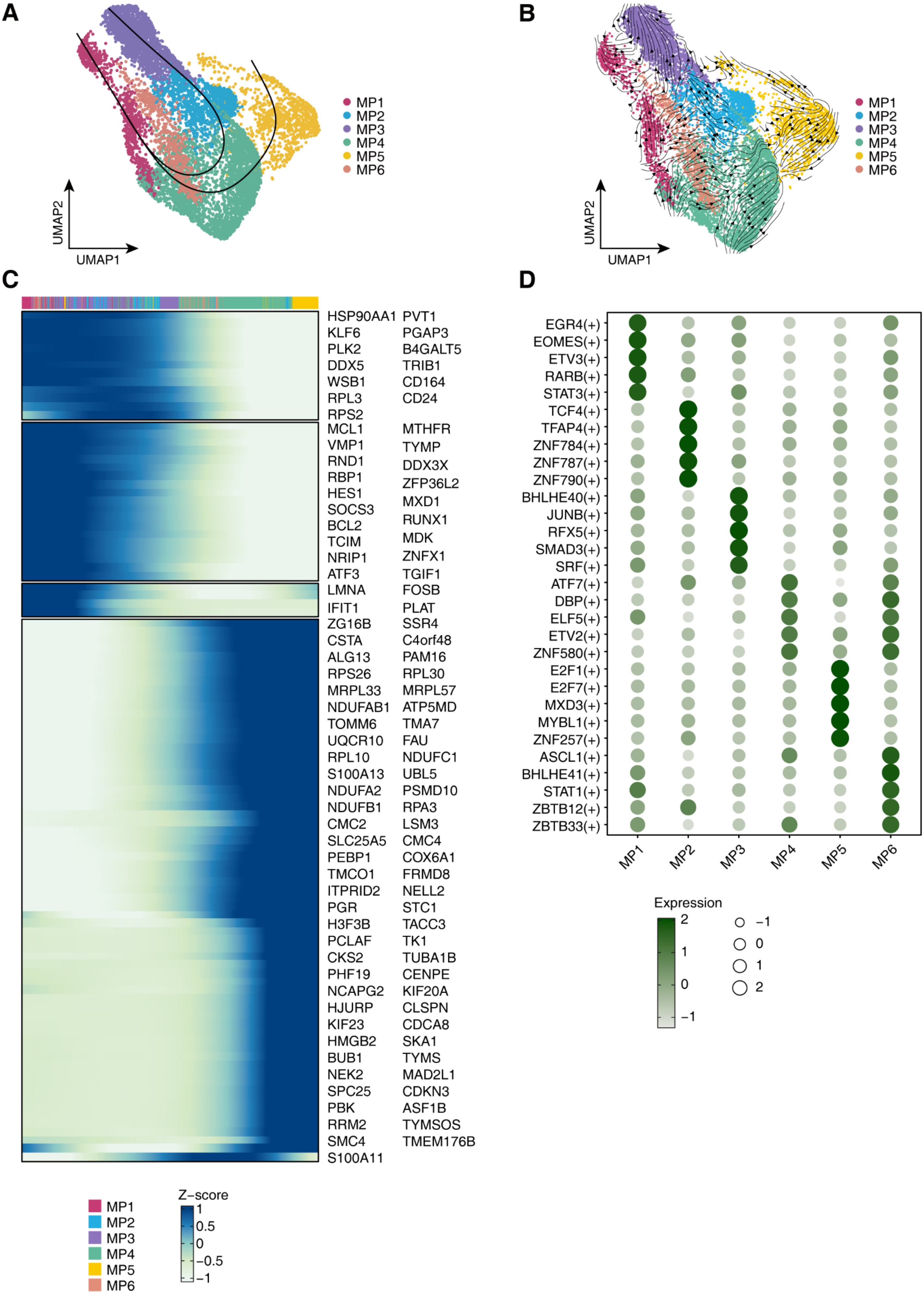
A
B
C
D
MP1
MP2
MP3
MP4
MP5
MP6
UMAP1
UMAP2
HSP90AA1 PVT1
KLF6 PGAP3
PLK2 B4GALT5
DDX5 TRIB1
WSB1 CD164
RPL3 CD24
RPS2
MCL1 MTHFR
VMP1 TYMP
RND1 DDX3X
RBP1 ZFP36L2
HES1 MXD1
SOCS3 RUNX1
BCL2 MDK
TCIM ZNFX1
NRIP1 TGIF1
ATF3 FOSB
LMNA PLAT
IFIT1 SSR4
ZG16B C4orf48
CSTA PAM16
ALG13 RPL30
RPS26 MRPL57
MRPL33 ATP5MD
NDUFAB1 TMA7
TOMM6 FAU
UQCR10 NDUFC1
RPL10 UBL5
S100A13 PSMD10
NDUFA2 RPA3
NDUFB1 LSM3
CMC2 CMC4
SLC25A5 COX6A1
PEBP1 FRMD8
TMCO1 NELL2
ITPRID2 STC1
PGR TACC3
H3F3B TK1
PCLAF TUBA1B
CKS2 CENPE
PHF19 KIF20A
NCAPG2 CLSPN
HJURP CDCA8
KIF23 SKA1
HMGB2 TYMS
BUB1 MAD2L1
NEK2 CDKN3
SPC25 ASF1B
PBK TYMSOS
RRM2 TMEM176B
SMC4
S100A11
Z−score
1
0.5
0
−0.5
−1
EGR4(+)
EOMES(+)
ETV3(+)
RARB(+)
STAT3(+)
TCF4(+)
TFAP4(+)
ZNF784(+)
ZNF787(+)
ZNF790(+)
BHLHE40(+)
JUNB(+)
RFX5(+)
SMAD3(+)
SRF(+)
ATF7(+)
DBP(+)
ELF5(+)
ETV2(+)
ZNF580(+)
E2F1(+)
E2F7(+)
MXD3(+)
MYBL1(+)
ZNF257(+)
ASCL1(+)
BHLHE41(+)
STAT1(+)
ZBTB12(+)
ZBTB33(+)
Expression
2
1
0
−1

Supplementary Figure 9. Trajectory and transcription factor analysis of MPs. Related to Figure 3.

(A) Pseudotime trajectory of malignant epithelial cells classified by MP identity. The black line indicates the inferred temporal trajectory. (B) RNA velocity plot of malignant epithelial cells classified by MP identity. (C) Heatmap showing genes with significant changes along pseudotime, with the top annotation indicating cells labeled by MP information. (D) Dot plot displaying the top five transcription factor activities for each malignant epithelial cell subpopulation.

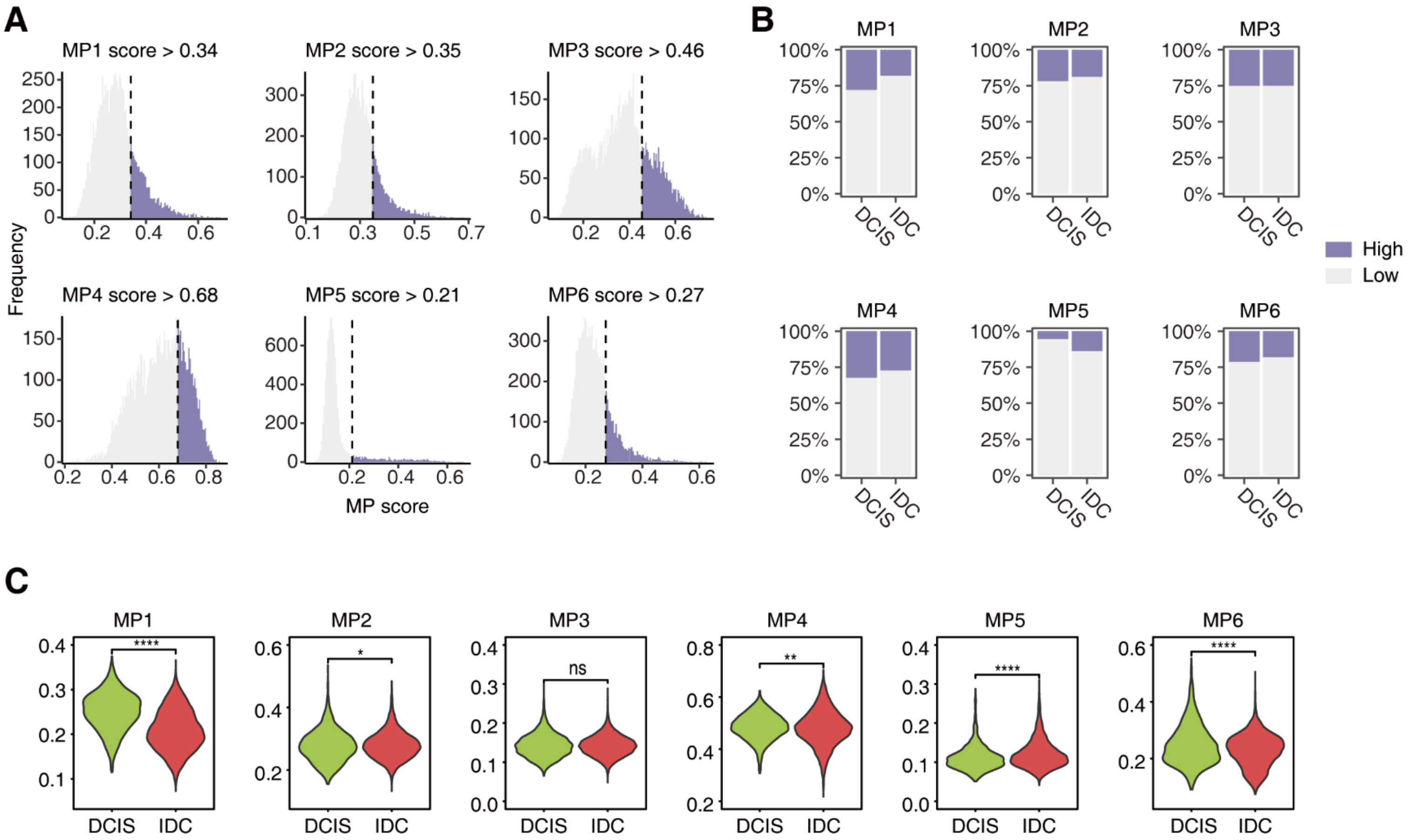

Supplementary Figure 10. The relationship between DCIS/IDC and MPs. Related to Figure 3.

(A) Spike plots showing the optimal cutoff values for MP scores in malignant epithelial cells from the scRNAseq data, with dashed vertical lines indicating the thresholds. (B) Proportional distributions of malignant epithelial cells classified according to MP scores in DCIS and IDC. (C) Violin plots comparing MP scores between DCIS and IDC in ST spots. Statistical significance was assessed using a two-sided Wilcoxon test. ****$P < 0.0001$, **$P < 0.01$, *$P < 0.05$, ns, not significant.

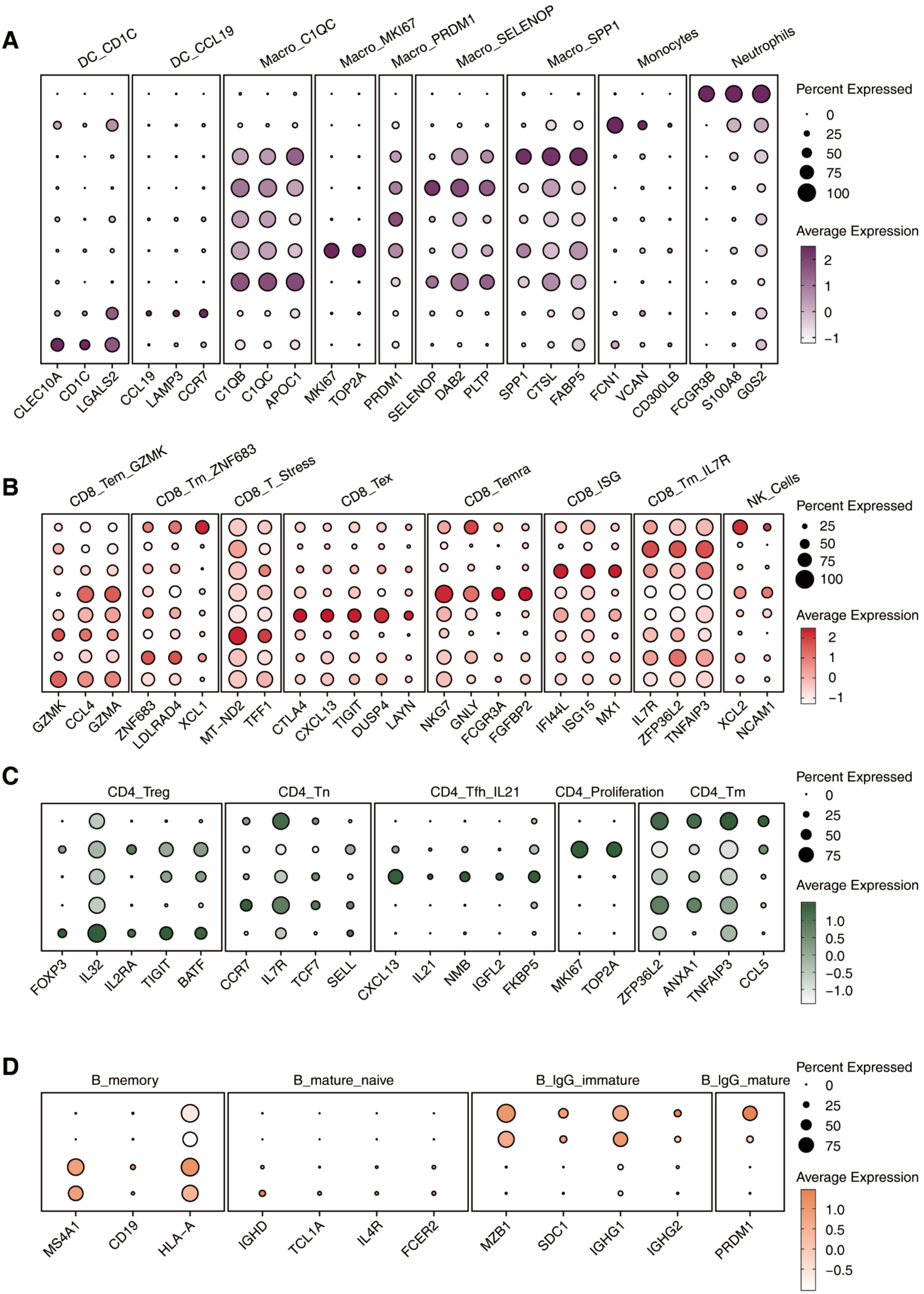
A
DC_CD1C
DC_CCL19
Macro_C1QC
Macro_MKI67
Macro_PRDM1
Macro_SELENOP
Macro_SPP1
Monocytes
Neutrophils
CLEC10A
CD1C
LGALS2
CCL19
LAMP3
CCR7
C1QB
C1QC
APOC1
MKI67
TOP2A
PRDM1
SELENOP
DAB2
PLTP
SPP1
CTSL
FABP5
FCN1
VCAN
CD300LB
FCGR3B
S100A8
G0S2
Percent Expressed
0
25
50
75
100
Average Expression
2
1
0
−1
B
CD8_Tem_GZMK
CD8_Tm_ZNF683
CD8_T_Stress
CD8_Tex
CD8_Temra
CD8_ISG
CD8_Tm_IL7R
NK_Cells
GZMK
CCL4
GZMA
ZNF683
LDLRAD4
XCL1
MT-ND2
TFF1
CTLA4
CXCL13
TIGIT
DUSP4
LAYN
NKG7
GNLY
FCGR3A
FGFBP2
IFI44L
ISG15
MX1
IL7R
ZFP36L2
TNFAIP3
XCL2
NCAM1
Percent Expressed
25
50
75
100
Average Expression
2
1
0
−1
C
CD4_Treg
CD4_Tn
CD4_Tfh_IL21
CD4_Proliferation
CD4_Tm
FOXP3
IL32
IL2RA
TIGIT
BATF
CCR7
IL7R
TCF7
SELL
CXCL13
IL21
NMB
IGFL2
FKBP5
MKI67
TOP2A
ZFP36L2
ANXA1
TNFAIP3
CCL5
Percent Expressed
0
25
50
75
Average Expression
1.0
0.5
0.0
−0.5
−1.0
D
B_memory
B_mature_naive
B_IgG_immature
B_IgG_mature
MS4A1
CD19
HLA-A
IGHD
TCL1A
IL4R
FCER2
MZB1
SDC1
IGHG1
IGHG2
PRDM1
Percent Expressed
0
25
50
75
Average Expression
1.0
0.5
0.0
−0.5

Supplementary Figure 11. Marker genes of immune cell subpopulations from scRNAseq. Related to Figure 4.

(A, B, C, D) Bubble plot visualizing the expression patterns of key marker genes across myeloid (A), CD8+T/NK (B), CD4+T (C), and B cell (D) subpopulations. The dot size corresponds to the fraction of cells expressing each gene, and the color intensity represents the normalized expression level.

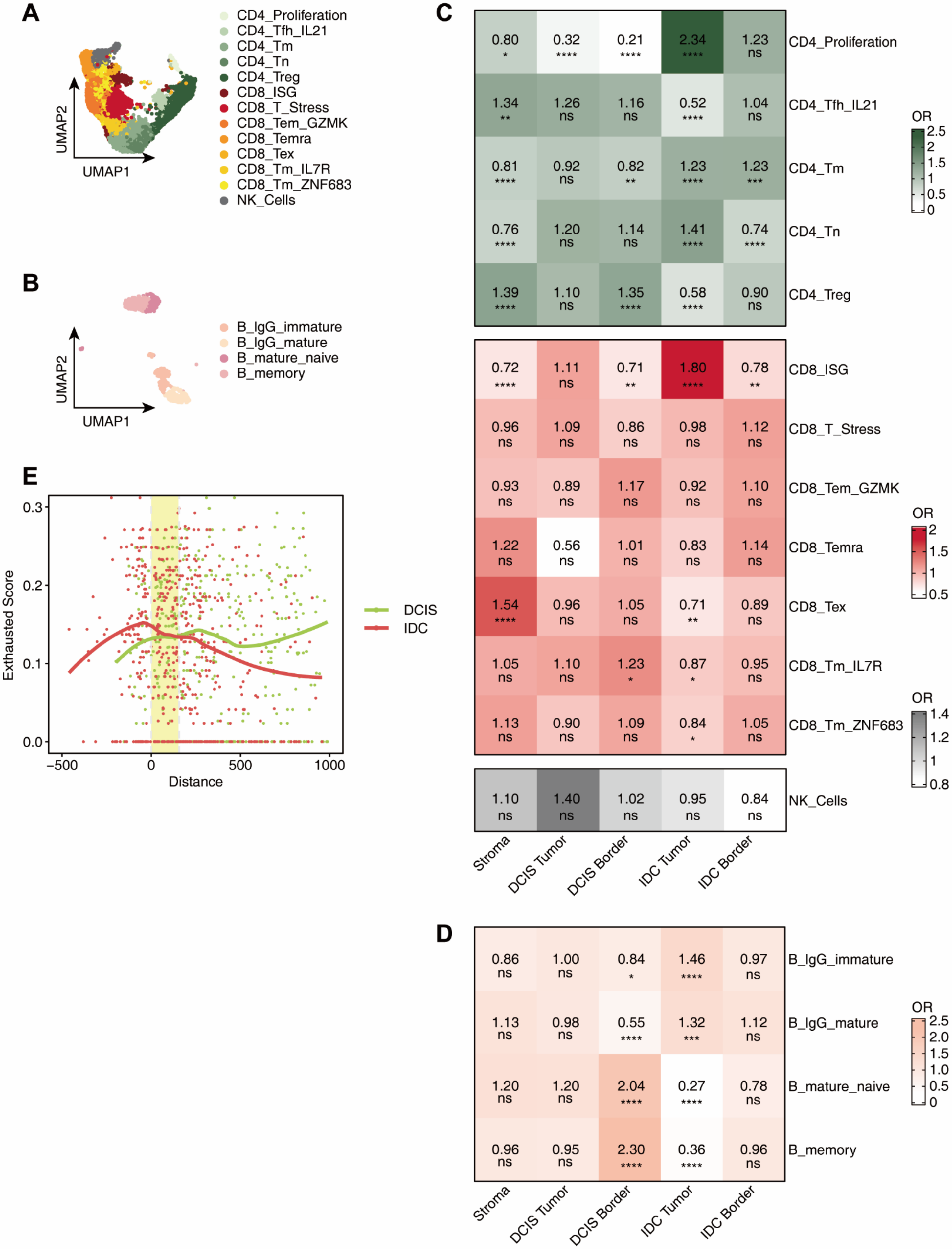

	Stroma	DCIS Tumor	DCIS Border	IDC Tumor	IDC Border
CD4_Proliferation	0.80 *	0.32 ****	0.21 ****	2.34 ****	1.23 ns
CD4_Tfh_IL21	1.34 **	1.26 ns	1.16 ns	0.52 ****	1.04 ns
CD4_Tm	0.81 ****	0.92 ns	0.82 **	1.23 ****	1.23 ***
CD4_Tn	0.76 ****	1.20 ns	1.14 ns	1.41 ****	0.74 ****
CD4_Treg	1.39 ****	1.10 ns	1.35 ****	0.58 ****	0.90 ns
CD8_ISG	0.72 ****	1.11 ns	0.71 **	1.80 ****	0.78 **
CD8_T_Stress	0.96 ns	1.09 ns	0.86 ns	0.98 ns	1.12 ns
CD8_Tem_GZMK	0.93 ns	0.89 ns	1.17 ns	0.92 ns	1.10 ns
CD8_Temra	1.22 ns	0.56 ns	1.01 ns	0.83 ns	1.14 ns
CD8_Tex	1.54 ****	0.96 ns	1.05 ns	0.71 **	0.89 ns
CD8_Tm_IL7R	1.05 ns	1.10 ns	1.23 *	0.87 *	0.95 ns
CD8_Tm_ZNF683	1.13 ns	0.90 ns	1.09 ns	0.84 *	1.05 ns
NK_Cells	1.10 ns	1.40 ns	1.02 ns	0.95 ns	0.84 ns

	Stroma	DCIS Tumor	DCIS Border	IDC Tumor	IDC Border
B_IgG_immature	0.86 ns	1.00 ns	0.84 *	1.46 ****	0.97 ns
B_IgG_mature	1.13 ns	0.98 ns	0.55 ****	1.32 ***	1.12 ns
B_mature_naive	1.20 ns	1.20 ns	2.04 ****	0.27 ****	0.78 ns
B_memory	0.96 ns	0.95 ns	2.30 ****	0.36 ****	0.96 ns

Supplementary Figure 12. Distribution patterns of T/NK and B cell subpopulations. Related to Figure 4.

(A) UMAP plot of T/NK cell subpopulations. (B) UMAP plot of B cell subpopulations. (C) ORs of T cell subpopulations occurring across different tissue regions. (D) ORs of B cell subpopulations occurring across different tissue regions. (E) Exhausted Score of CD8_Tex cells based on distance from the DCIS and IDC boundary. 0 represents the boundary, with positive values indicating regions outside the boundary and negative values indicating regions inside. The yellow dashed box highlights the 0-200 μm border area. Statistical significance was assessed using Fisher's exact test in (C) and (D), with adjustment for multiple comparisons using the BH method; ****$P < 0.0001$, ***$P < 0.001$, **$P < 0.01$, * $P < 0.05$, ns, not significant.

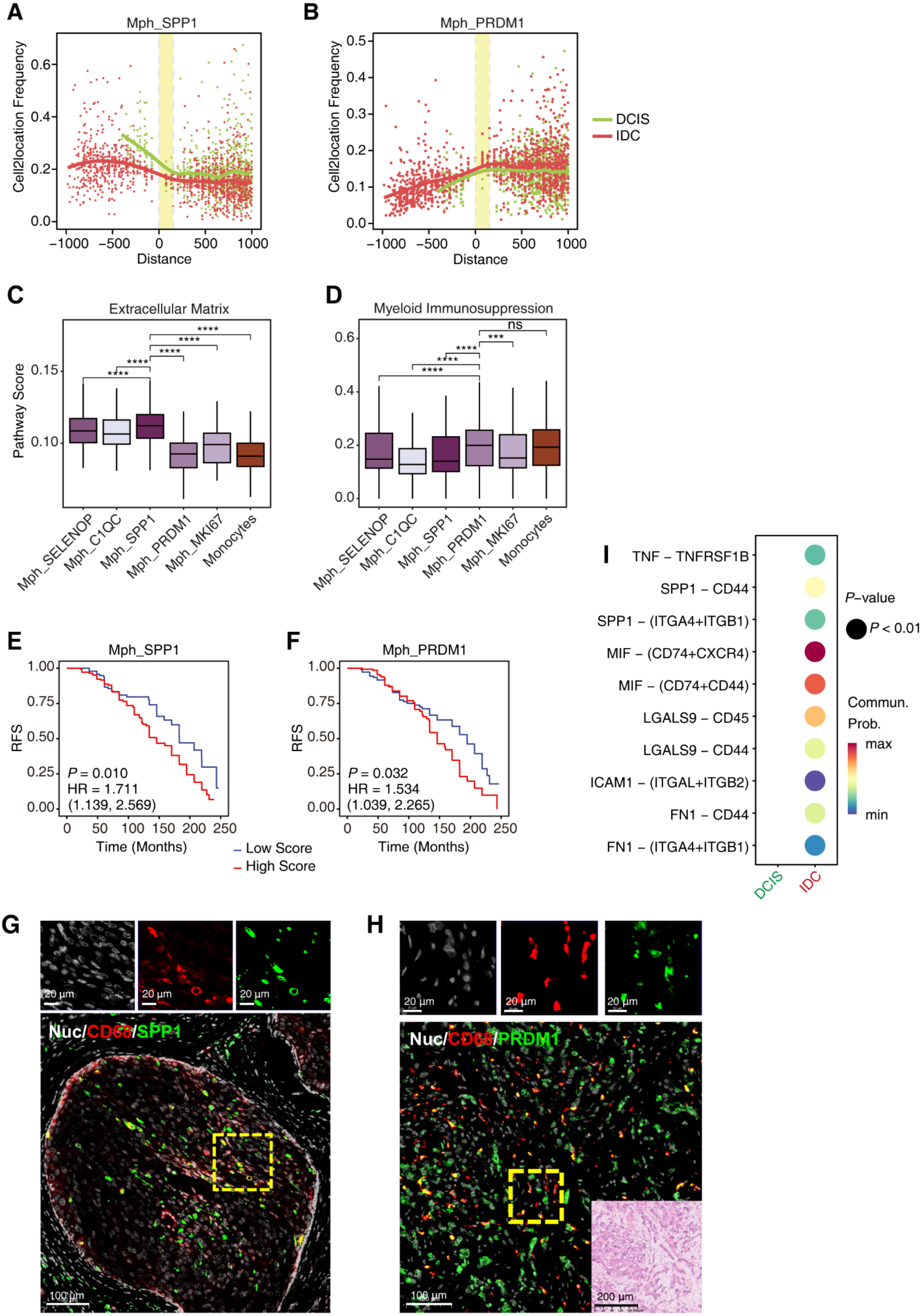

A
Mph_SPP1
Cell2location Frequency
Distance
B
Mph_PRDM1
DCIS
IDC
C
Extracellular Matrix
Pathway Score
D
Myeloid Immunosuppression
ns
Mph_SELENOP
Mph_C1QC
Mph_SPP1
Mph_PRDM1
Mph_MKI67
Monocytes
E
Mph_SPP1
RFS
P = 0.010
HR = 1.711
(1.139, 2.569)
Time (Months)
Low Score
High Score
F
Mph_PRDM1
P = 0.032
HR = 1.534
(1.039, 2.265)
I
TNF – TNFRSF1B
SPP1 – CD44
SPP1 – (ITGA4+ITGB1)
MIF – (CD74+CXCR4)
MIF – (CD74+CD44)
LGALS9 – CD45
LGALS9 – CD44
ICAM1 – (ITGAL+ITGB2)
FN1 – CD44
FN1 – (ITGA4+ITGB1)
P–value
P < 0.01
Commun. Prob.
max
min
G
Nuc/CD68/SPP1
20 µm
100 µm
H
Nuc/CD68/PRDM1
200 µm

Supplementary Figure 13. Distribution and functional patterns of myeloid cell subpopulations. Related to Figure 4.

(A, B) Frequency distribution of Mph_SPP1 and Mph_PRDM1 estimated by cell2location as a function of distance from the DCIS and IDC boundary, calculated as described in Supplementary Figure 12E. (C, D) Box plots displaying “Extracellular Matrix” and “Myeloid Immunosuppression” pathway scores in macrophages and monocytes. Statistical significance was assessed using a two-sided Wilcoxon test; ****$P < 0.0001$, ***$P < 0.001$, ns, not significant. (E, F) Kaplan-Meier survival curves illustrating RFS outcomes associated with Mph_SPP1 and Mph_PRDM1 in the HTAN database. Significance was assessed using the log-rank test. HR and 95% CI were estimated using univariable Cox proportional hazards regression. (G, H) Representative mIHC staining images of CD68 with SPP1 in DCIS and CD68 with PRDM1 in IDC. The inset shows the corresponding H&E image. (I) Top 10 upregulated ligand-receptor pairs between Mph_PRDM1 and CD8_Tex in IDC compared with DCIS. Dot size represents statistical significance.

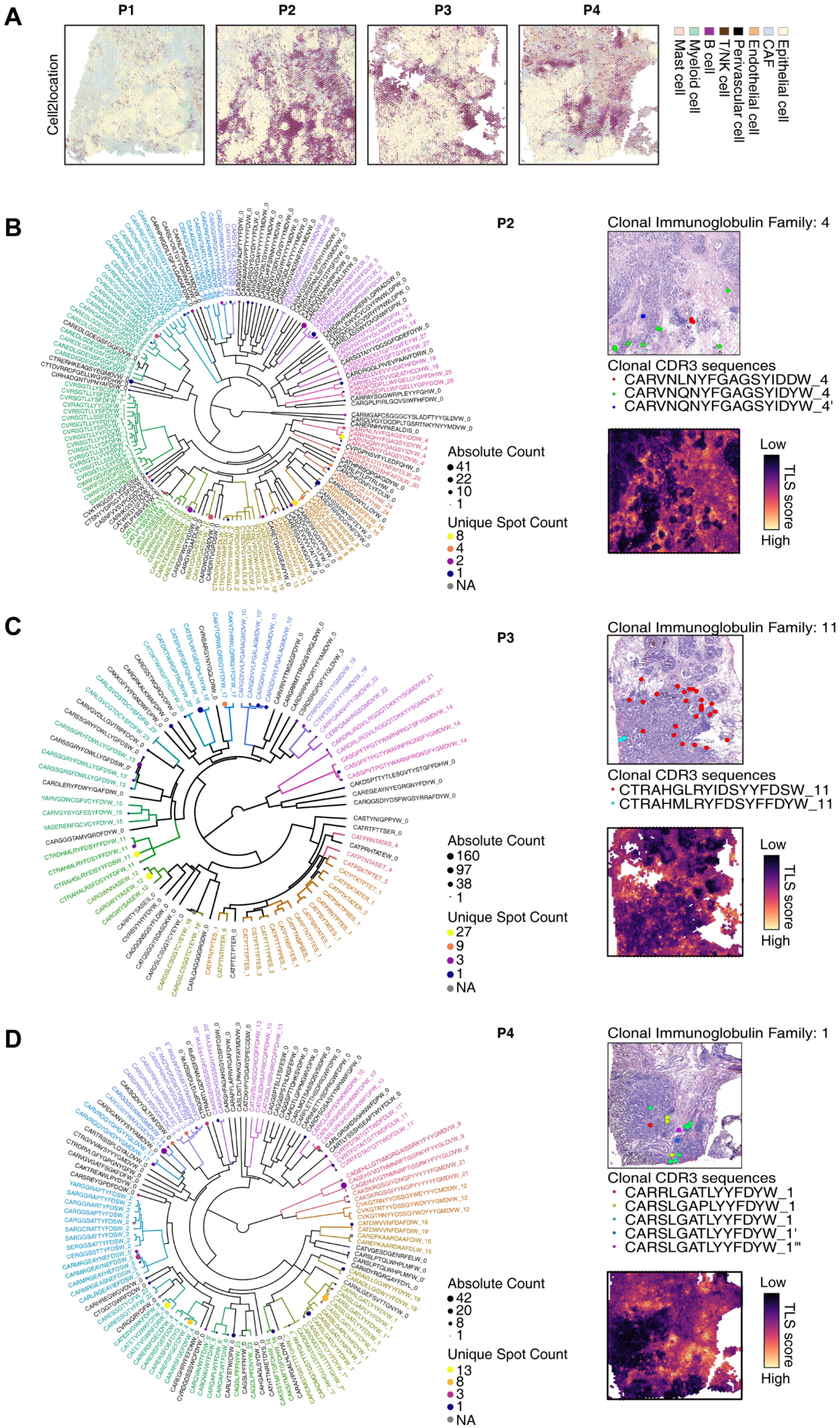
A
P1
P2
P3
P4
Cell2location
Epithelial cell
CAF
Endothelial cell
Perivascular cell
T/NK cell
B cell
Myeloid cell
Mast cell
B
P2
Clonal Immunoglobulin Family: 4
Clonal CDR3 sequences
CARVNLNYFGAGSYIDDW_4
CARVNQNYFGAGSYIDYW_4
CARVNQNYFGAGSYIDYW_4'
Absolute Count
41
22
10
1
Unique Spot Count
8
4
2
1
NA
Low
TLS score
High
C
P3
Clonal Immunoglobulin Family: 11
Clonal CDR3 sequences
CTRAHGLRYIDSYYFDSW_11
CTRAHMLRYFDSYFFDYW_11
Absolute Count
160
97
38
1
Unique Spot Count
27
9
3
1
NA
Low
TLS score
High
D
P4
Clonal Immunoglobulin Family: 1
Clonal CDR3 sequences
CARRLGATLYYFDYW_1
CARSLGAPLYYFDYW_1
CARSLGATLYYFDYW_1
CARSLGATLYYFDYW_1'
CARSLGATLYYFDYW_1'''
Absolute Count
42
20
8
1
Unique Spot Count
13
8
3
1
NA
Low
TLS score
High

Supplementary Figure 14. BCR analysis of spatial transcriptomics. Related to Figure 4.

(A) Cell type proportions estimated by cell2location in ST datasets. (B, C, D) BCR profiles of P2, P3 and P4. Left plot shows CDR3 distance trees illustrating the clonal relationships among immunoglobulin heavy chain (IGH) clonotypes. Each clonotype is represented by its CDR3 amino acid sequence and assigned to a specific clonal family (0 indicates non-clonal sequences). Clonal families are highlighted with distinct branch colors and text labels, while black represents unique sequences. The dot size corresponds to the abundance of each clonotype, and the dot color indicates the number of spatial spots in which the clonotype is detected. The top right plot shows spatial mapping of IGH clonotypes from selected clonal families. The bottom right plot shows spatial expression patterns of the TLS imprint signature.

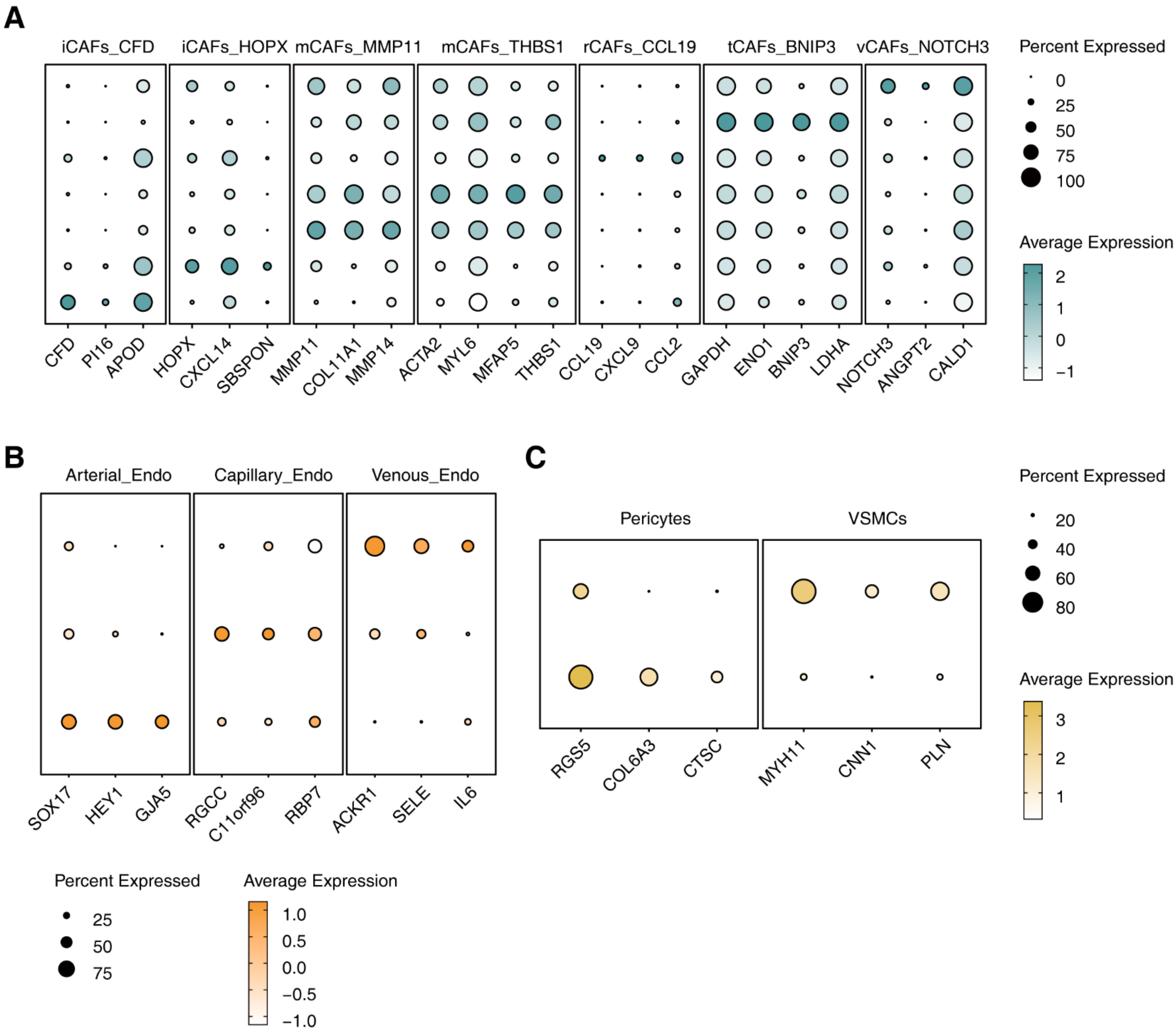

Supplementary Figure 15. Marker genes of stroma subpopulations from scRNAseq. Related to Figure 5.

(A, B, C) Bubble plot visualizing the expression patterns of key marker genes across CAF (A), endothelial (B) and perivascular (C) subpopulations. The dot size corresponds to the fraction of cells expressing each gene, while the color intensity represents the normalized expression level.

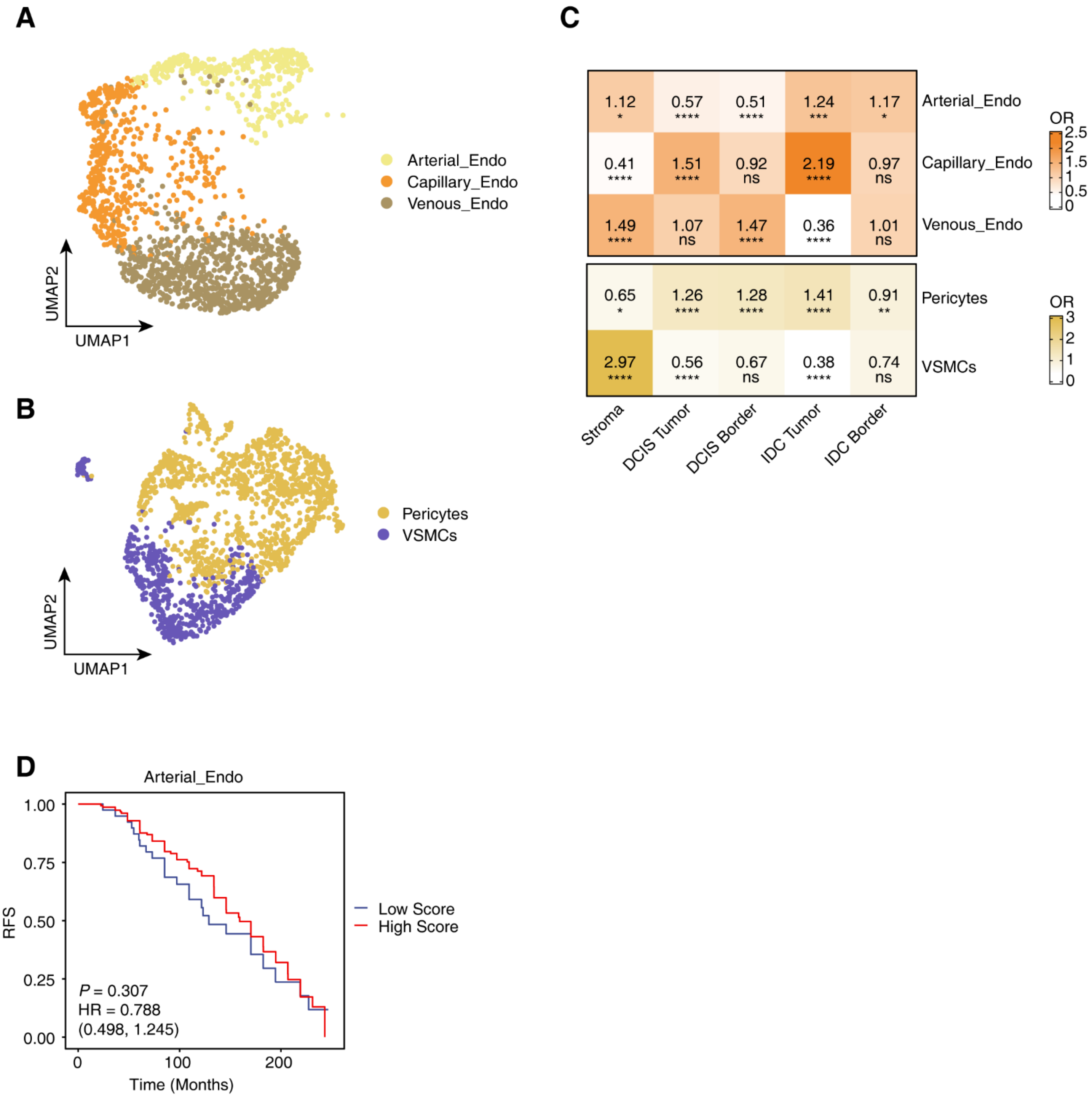

Supplementary Figure 16. Distribution patterns of endothelial and perivascular subpopulations. Related to Figure 5.

(A) UMAP plot of endothelial cell subpopulations. (B) UMAP plot of perivascular subpopulations. Vascular smooth muscle cells, VSMCs. (C) ORs of endothelial and perivascular subpopulations occurring across different tissue regions. Statistical significance was assessed using Fisher's exact test, with adjustment for multiple comparisons using the BH method; $****P < 0.0001$, $***P < 0.001$, $**P < 0.01$, $*P < 0.05$, ns, not significant. (D) Kaplan-Meier survival curve illustrating RFS outcomes associated with the Arterial_Endo subpopulation in the HTAN database. Statistical significance was assessed using a log-rank test. HR and 95% CI were estimated using univariable Cox proportional hazards regression.

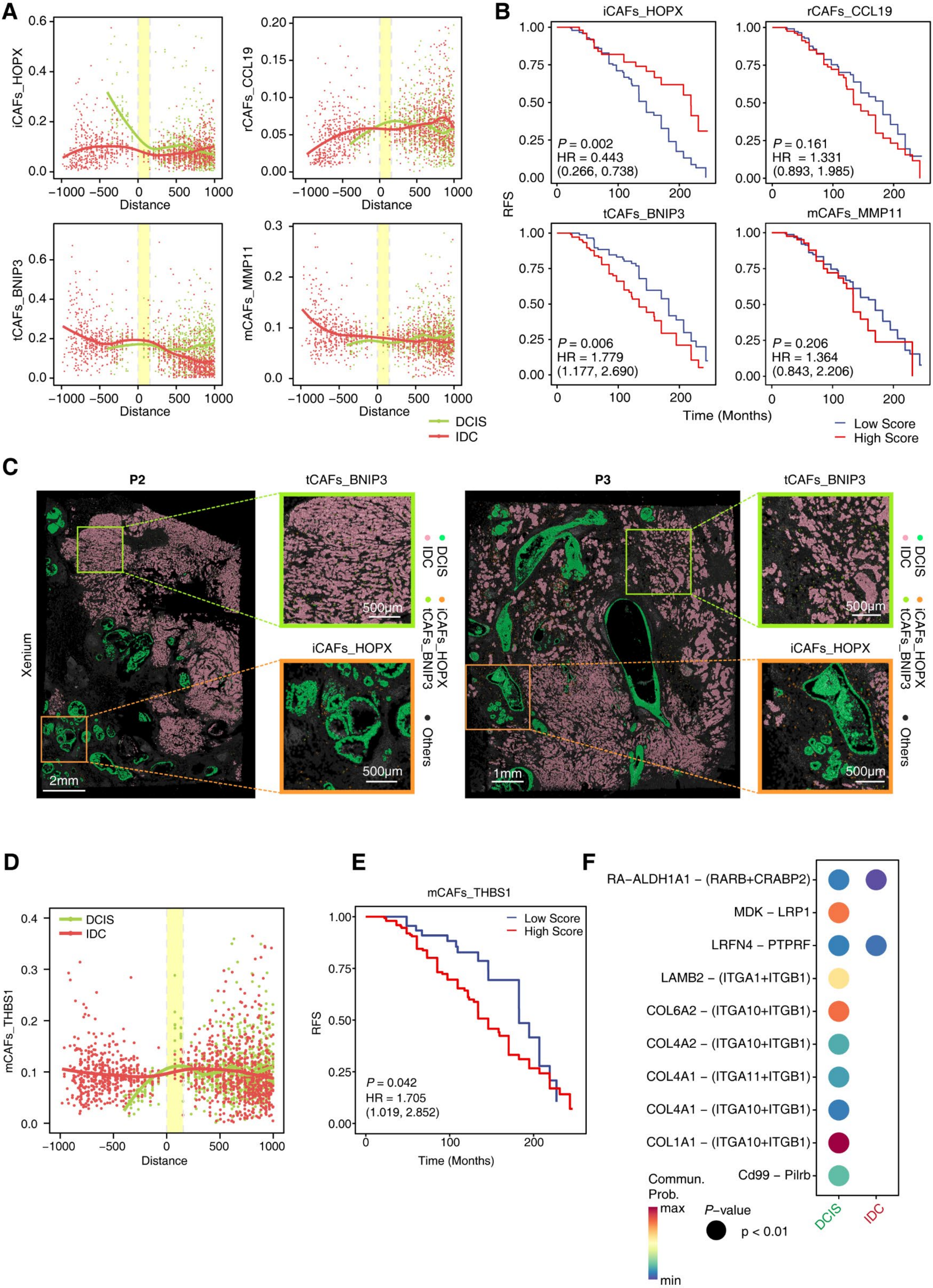

A
iCAFs_HOPX
rCAFs_CCL19
tCAFs_BNIP3
mCAFs_MMP11
Distance
DCIS
IDC
B
iCAFs_HOPX
P = 0.002
HR = 0.443
(0.266, 0.738)
rCAFs_CCL19
P = 0.161
HR = 1.331
(0.893, 1.985)
tCAFs_BNIP3
P = 0.006
HR = 1.779
(1.177, 2.690)
mCAFs_MMP11
P = 0.206
HR = 1.364
(0.843, 2.206)
RFS
Time (Months)
Low Score
High Score
C
P2
P3
Xenium
tCAFs_BNIP3
iCAFs_HOPX
DCIS
IDC
iCAFs_HOPX
tCAFs_BNIP3
Others
2mm
1mm
500μm
D
mCAFs_THBS1
Distance
DCIS
IDC
E
mCAFs_THBS1
Low Score
High Score
P = 0.042
HR = 1.705
(1.019, 2.852)
RFS
Time (Months)
F
RA-ALDH1A1 – (RARB+CRABP2)
MDK – LRP1
LRFN4 – PTPRF
LAMB2 – (ITGA1+ITGB1)
COL6A2 – (ITGA10+ITGB1)
COL4A2 – (ITGA10+ITGB1)
COL4A1 – (ITGA11+ITGB1)
COL4A1 – (ITGA10+ITGB1)
COL1A1 – (ITGA10+ITGB1)
Cd99 – Pilrb
Commun. Prob.
max
min
P–value
p < 0.01
DCIS
IDC

Supplementary Figure 17. Distribution patterns of CAF subpopulations. Related to Figure 5.

(A) Frequency distributions of iCAFs_HOPX, rCAFs_CCL19, tCAFs_BNIP3, and mCAFs_MMP11 estimated by cell2location as a function of distance from the DCIS and IDC boundary, as described in Supplementary Figure 12E. (B) Kaplan-Meier survival curves illustrating RFS outcomes associated with these CAF subpopulations in the HTAN database. (C) Xenium spatial transcriptomic visualization of DCIS, IDC, tCAFs_BNIP3, and iCAFs_HOPX, with zoomed-in views of the ROI for tCAFs_BNIP3 and iCAFs_HOPX shown on the right. (D) Frequency distribution of mCAFs_THBS1 estimated by cell2location as a function of distance from the DCIS and IDC boundary, as described in Supplementary Figure 12E. (E) Kaplan-Meier survival curve illustrating RFS outcomes associated with the mCAFs_THBS1 subpopulation in the HTAN database. Statistical significance in (B) and (E) was assessed using a log-rank test. HR and 95% CI were estimated using univariable Cox proportional hazards regression. (F) Top 10 upregulated ligand-receptor pairs between mCAFs_THBS1 and myoepithelial cells in DCIS and IDC. Dot size represents statistical significance.

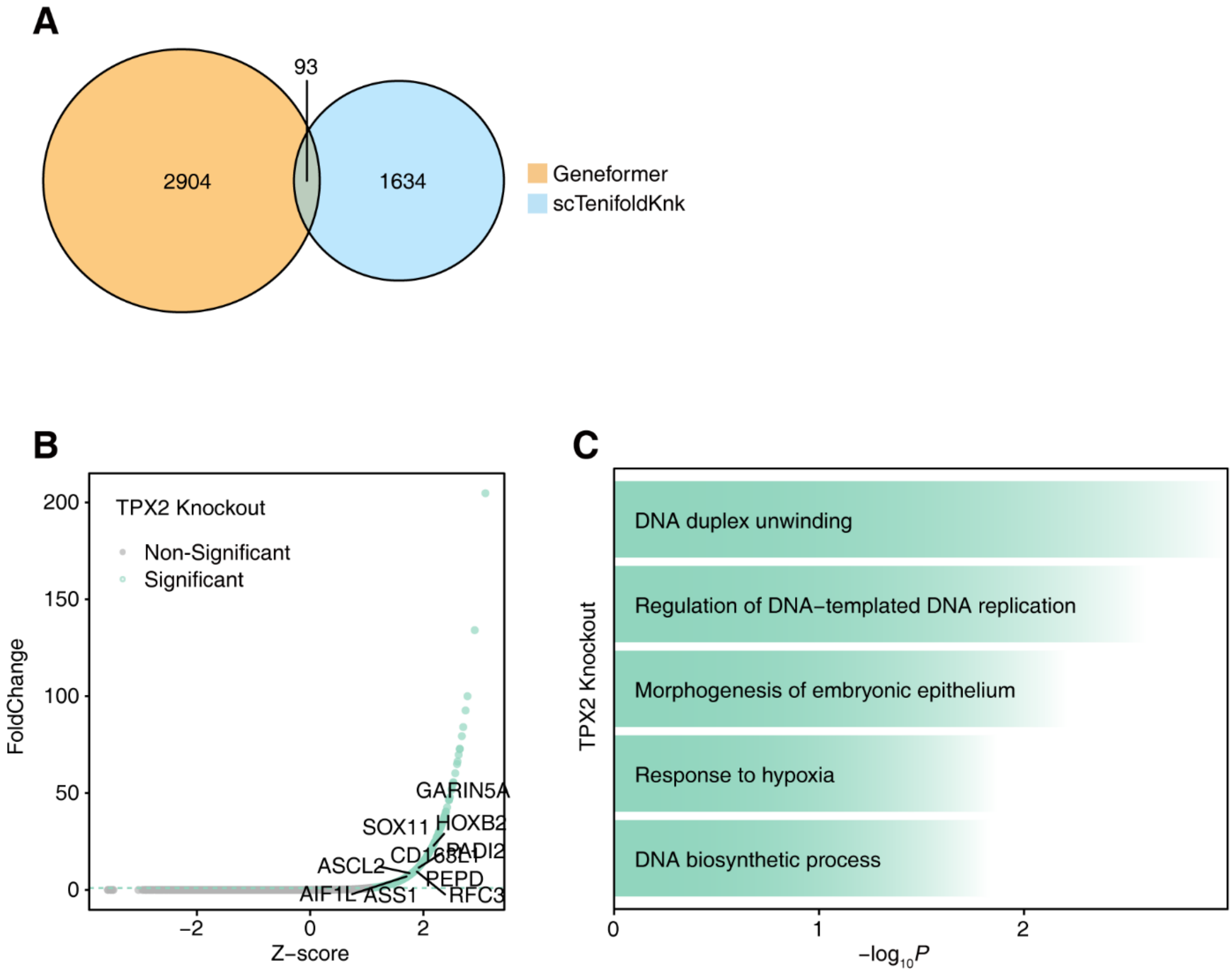

Supplementary Figure 18. Transcriptional perturbations following virtual knockout of *TPX2*. Related to Figure 7.

(A) Venn diagram indicates the overlap of perturbed genes identified by scTenifoldKnk and Geneformer after virtual knockout of *TPX2* in scRNAseq data from the HTAN pure DCIS cohort. (B) Dot plot showing the magnitude of gene changes after virtual knockout of *TPX2*, highlighting the top 10 shared perturbed genes identified by both scTenifoldKnk and Geneformer. (C) GO pathway enrichment analysis of significantly perturbed genes shared by scTenifoldKnk and Geneformer following virtual knockout of *TPX2*.
